# Donor‐Cell Senescence Redirects PINK1‐Marked Mitochondrial Cargo From Mitophagy to Microvesicle Transfer Through SUMO3‐Stabilized HSPA9

**DOI:** 10.1002/advs.78392

**Published:** 2026-10-25

**Authors:** Zixuan Ou, Mingke Zhao, Zhiwei Ling, Lianqi Peng, Bide Tong, Huaizhen Liang, Hongchuan Wang, Di Wu, Yifan Du, Zhi Du, Shihao Zhang, Yuyuan Jia, Yifan Qian, Gan Xu, Liuliang He, Gang Li, Shun Zhang, Lei Tan, Kun Wang, Xiaobo Feng, Cao Yang, Zhiwei Liao

**Affiliations:** ^1^ Department of Orthopaedics Union Hospital Tongji Medical College Huazhong University of Science and Technology Wuhan People's Republic of China; ^2^ Department of Dermatology Union Hospital Tongji Medical College Huazhong University of Science and Technology Wuhan People's Republic of China

**Keywords:** cellular senescence, intervertebral disc degeneration, microvesicles, mitochondrial transfer, SUMOylation

## Abstract

Extracellular vesicle therapies have been developed for tissue repair, but how donor‐cell senescence reshapes vesicle‐mediated mitochondrial transfer remains unclear. Here, we show that senescent adipose‐derived stem cells (ADSCs) redirect damaged mitochondria into microvesicles (MVs), converting protective vesicles into pro‐degenerative carriers. Senescent ADSC‐derived MVs are enriched in PINK1‐marked, oxidatively damaged mitochondrial cargo. After transfer to nucleus pulposus cells (NPCs), this cargo disrupts recipient mitochondrial homeostasis, activates the cGAS‐STING‐NLRP3 inflammatory axis, and aggravates intervertebral disc degeneration. Proteomic and functional analyses identify HSPA9 as a mitochondrial cargo‐sorting adaptor. Senescent stress induces SUMO3 conjugation of HSPA9 at K612, stabilizing HSPA9 by limiting ubiquitin‐proteasomal degradation. Stabilized HSPA9 uses its peptide‐binding domain to engage the kinase domain of activated PINK1 and links PINK1‐positive damaged mitochondria to Annexin A1‐associated MV structures. This SUMO3‐HSPA9‐PINK1 module diverts damaged mitochondria from PINK1‐Parkin mitophagy toward MV‐mediated mitochondrial transfer. Donor‐cell reprogramming that restrains the release of damaged mitochondria and reinforces mitophagic clearance restores the in vivo therapeutic efficacy of senescent ADSC‐derived MVs. These findings identify mitochondrial cargo fate as a switch between vesicle‐mediated repair and degeneration.

## 1 Introduction

Intercellular transfer of mitochondrial material is an important route by which donor cells influence recipient‐cell metabolism, stress responses, and inflammatory state. Extracellular vesicles (EVs), including microvesicles (MVs), can carry mitochondrial proteins, mitochondrial DNA (mtDNA), mitochondrial fragments and mitochondria‐containing structures [1, 2, 3]. The biological consequence of this transfer depends on cargo quality. Functional mitochondrial cargo may support recipient‐cell bioenergetics, whereas damaged mitochondrial cargo can deliver mitochondrial danger‐associated molecular patterns (DAMPs), increase oxidative stress, and activate innate immune signaling [4]. Thus, vesicle‐mediated mitochondrial transfer should not be viewed as intrinsically reparative; under chronic stress or senescence, the same route may propagate organelle injury.

This cargo‐quality problem is particularly relevant to intervertebral disc degeneration (IVDD). Nucleus pulposus cells (NPCs) rely on mitochondrial homeostasis to maintain survival, matrix metabolism, and resistance to oxidative stress [5, 6]. During IVDD, mitochondrial dysfunction, mtDNA leakage, and sterile inflammation are closely linked to NPC senescence, matrix degradation, and disc structural failure [7]. Cytosolic mtDNA can activate cGAS‐STING signaling and subsequently engage the NLRP3 inflammasome, converting mitochondrial damage into inflammatory degeneration [6, 8]. Clinically, IVDD is a major contributor to chronic low back pain, which remains a leading cause of disability worldwide [9, 10]. Therefore, identifying how mitochondrial damage signals are transferred to NPCs may help explain how disc degeneration is amplified and how vesicle‐based therapies fail or succeed.

Adipose‐derived stem cell (ADSC)‐derived vesicles are being explored as cell‐free therapeutics for IVDD because they can regulate NPC oxidative stress, inflammation, cell death, and matrix metabolism [11, 12]. Recent evidence that mesenchymal stem cell‐derived mitochondria‐containing MVs improve NPC mitochondrial function further suggests that mitochondrial transfer contributes to vesicle‐mediated disc repair [13, 14]. However, this therapeutic model assumes that vesicles deliver beneficial cargo. Donor‐cell senescence may challenge this assumption. Senescent cells remodel EV secretion and cargo composition, and senescence‐associated vesicles can transmit stress signals to neighboring cells [15, 16]. In the context of ADSC‐derived MV therapy, the key issue is therefore not only whether MVs are taken up by NPCs, but whether they transfer functional or damaged mitochondrial cargo.

Damaged mitochondria are classically handled through PINK1‐Parkin mitophagy [17]. Upon mitochondrial depolarization, PINK1 accumulates on the outer mitochondrial membrane and promotes Parkin‐dependent autophagic clearance of injured mitochondria [17, 18, 19]. Whether PINK1‐marked mitochondria can instead be redirected into extracellular MV export under senescent stress remains unclear. Such a route would convert a donor‐cell mitochondrial quality‐control defect into an intercellular transfer mechanism for mitochondrial DAMPs. The molecular adaptor that couples damaged mitochondria to MV biogenesis, and how this sorting decision determines MV function, are unresolved.

Here, we show that donor‐cell senescence redirects mitochondrial transfer by ADSC‐derived MVs. Normal ADSC‐derived MVs (MV_normal_) protect NPC mitochondrial homeostasis and suppress inflammatory signaling, whereas senescent ADSC‐derived MVs (MV_stress_) become enriched in PINK1‐marked, oxidatively damaged mitochondrial cargo. After transfer to NPCs, this cargo activates the cGAS‐STING‐NLRP3 inflammatory axis and aggravates IVDD. Mechanistically, we identify HSPA9 as a mitochondrial cargo‐sorting adaptor. Senescent stress induces SUMO3 conjugation of HSPA9 at K612, stabilizing HSPA9 by limiting ubiquitin‐proteasome‐dependent degradation. Stabilized HSPA9 uses its peptide‐binding domain to engage the kinase domain of activated PINK1 and links PINK1‐positive damaged mitochondria to Annexin A1‐associated MV structures. This SUMO3‐HSPA9‐PINK1 module shifts damaged mitochondria away from PINK1‐Parkin mitophagy and toward MV‐mediated transfer. Conversely, donor‐cell reprogramming that restrains damaged mitochondrial release and reinforces mitophagic clearance restores the anti‐inflammatory and disc‐protective efficacy of MV_stress_ (Scheme 1). These findings define mitochondrial cargo fate as a checkpoint determining whether ADSC‐derived MVs repair degenerating discs or propagate mitochondrial inflammatory injury.

**SCHEME 1:**
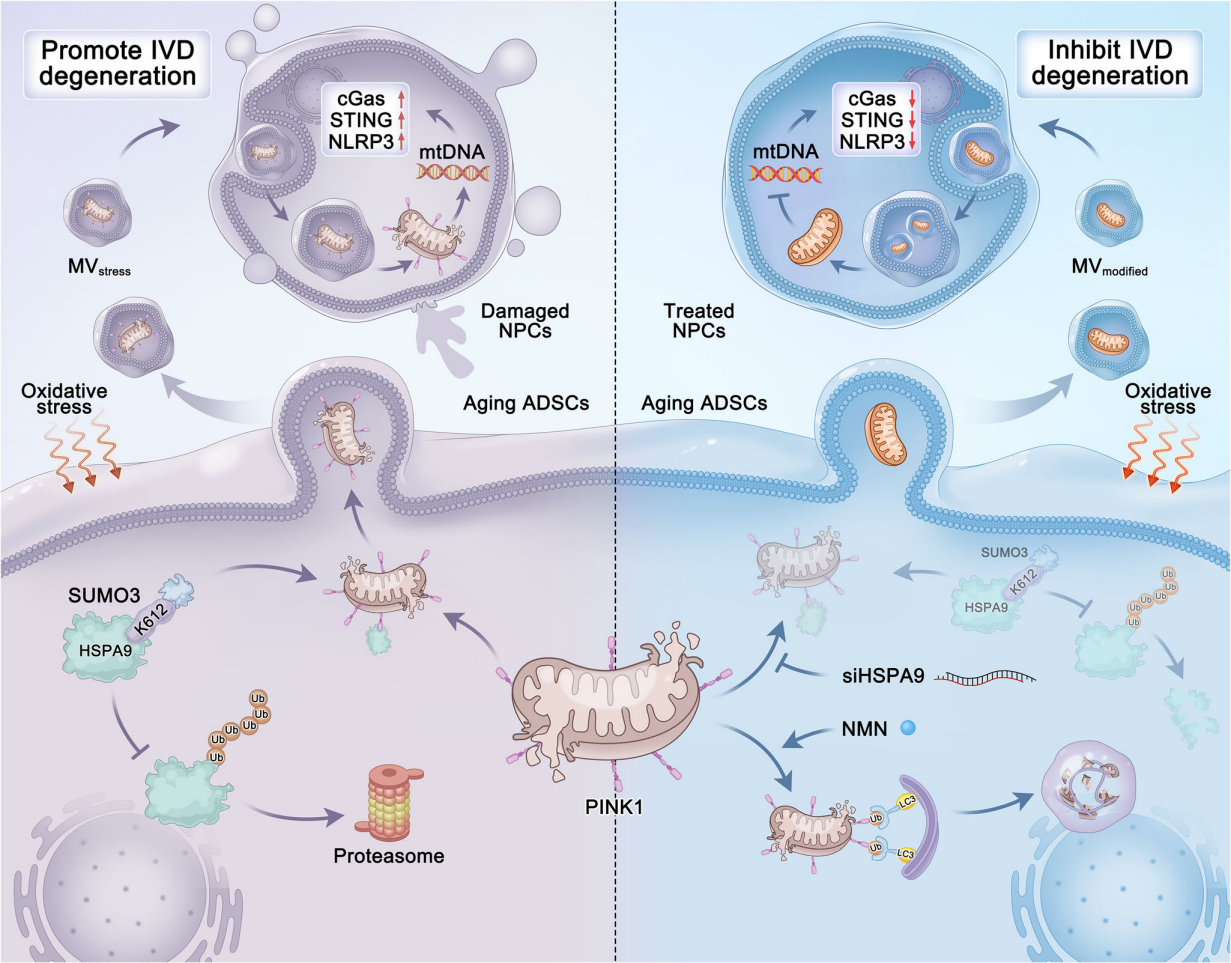
Donor‐cell mitochondrial cargo sorting determines the pathological or therapeutic identity of ADSC‐derived MVs in IVDD. Senescent stress promotes SUMO3‐dependent stabilization of HSPA9 and facilitates HSPA9‐mediated loading of PINK1‐marked damaged mitochondrial cargo into MVs. Transfer of this cargo to recipient NPCs disrupts mitochondrial homeostasis and activates the cGAS‐STING‐NLRP3 inflammatory axis. Donor‐cell reprogramming through NMN treatment and HSPA9 silencing limits damaged mitochondrial cargo export and restores the protective activity of ADSC‐derived MVs.

## 2 Results

### 2.1 Senescent ADSC‐Derived MVs Lose Their Protective Effects and Aggravate Mitochondrial Inflammatory Injury in NPCs

To investigate how ADSC‐derived MVs regulate NPC injury, we first isolated ADSCs from human adipose tissues and characterized their phenotype (Figure S1a). The isolated cells displayed the typical spindle‐shaped, adherent morphology of ADSCs. Flow cytometry showed high expression of the mesenchymal stem cell (MSC) markers CD73, CD90, and CD105 and low expression of human leukocyte antigen‐DR (HLA‐DR) (Figure S1b–e). Alizarin Red S and Oil Red O staining further confirmed osteogenic and adipogenic differentiation potential, respectively, consistent with the basic phenotypic properties of human ADSCs [20] (Figure S1f). We then isolated MVs from ADSC‐conditioned medium by sequential centrifugation and characterized their morphology, size distribution, and particle concentration using transmission electron microscopy (TEM) and nanoparticle tracking analysis (NTA) [21]. TEM showed typical round or oval membrane‐bound vesicles. NTA further indicated that ADSC‐derived MVs were mainly distributed over the 200 nm range and had an original particle concentration of 9.3 × 10^10^ particles/mL (Figure 1a–c). This size distribution is comparable to previous characterization of MSC‐derived MVs [13, 22]. Together, the isolation workflow, TEM morphology, and NTA‐based particle analysis confirmed the successful generation of ADSC‐derived MV fractions for subsequent functional assays.

**FIGURE 1:**
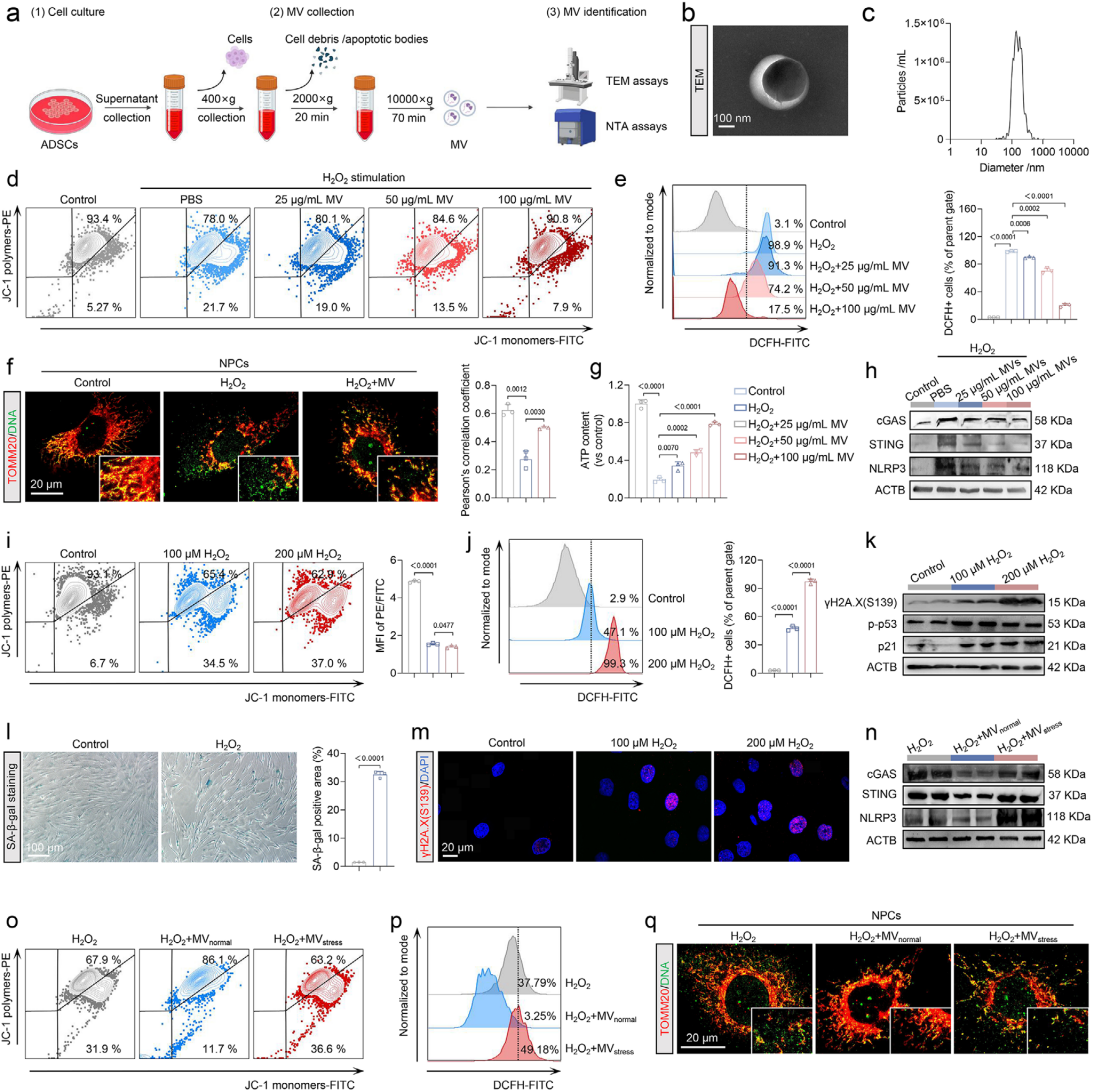
Senescent ADSC‐derived MVs lose the protective effects of normal ADSC‐derived MVs and aggravate mitochondrial inflammatory injury in NPCs. (a) Schematic illustration of MV isolation from ADSC‐conditioned medium by sequential centrifugation; (b) Representative TEM image of ADSC‐derived MVs. Scale bar, 100 nm; (c) NTA showing the size distribution of isolated ADSC‐derived MVs; (d) JC‐1 flow cytometry analysis of mitochondrial membrane potential in H_2_O_2_‐injured NPCs treated with different concentrations of MV_normal_; (e) DCFH flow cytometry analysis of intracellular ROS levels in H_2_O_2_‐injured NPCs treated as indicated; (f) Representative IF images showing TOMM20 and DNA in NPCs, with quantification of mitochondrial‐DNA colocalization. Scale bar, 20 µm; (g) ATP content in H_2_O_2_‐injured NPCs treated with different concentrations of MV_normal_; (h) WB analysis of cGAS, STING and NLRP3 in H_2_O_2_‐injured NPCs treated with different concentrations of MV_normal_; (i) JC‐1 flow cytometry analysis of mitochondrial membrane potential in ADSCs exposed to increasing concentrations of H_2_O_2;_ (j) DCFH flow cytometry analysis of ROS accumulation in ADSCs exposed to increasing concentrations of H_2_O_2;_ (k) WB analysis of γH2A.X(S139), p‐p53 and p21 in ADSCs exposed to increasing concentrations of H_2_O_2_. (l) SA‐β‐gal staining of ADSCs treated with H_2_O_2_. Scale bar, 100 µm; (m) IF staining of γH2A.X(S139) in ADSCs treated with increasing concentrations of H_2_O_2_. Scale bar, 20 µm; (n) WB analysis of cGAS, STING and NLRP3 in H_2_O_2_‐injured NPCs treated with MV_normal_ or MV_stress_; (o) JC‐1 flow cytometry analysis of mitochondrial membrane potential in H_2_O_2_‐injured NPCs treated with MV_normal_ or MV_stress_; (p) DCFH flow cytometry analysis of ROS levels in H_2_O_2_‐injured NPCs treated as indicated; (q) Representative IF images showing TOMM20 and DNA in H_2_O_2_‐injured NPCs treated with MV_norma_l or MV_stress_. Scale bar, 20 µm. Data are presented as mean ± s.d. from n = 3 biological replicates obtained from three independent experiments. Technical replicates were averaged within each biological replicate and were not treated as independent n values. Statistical significance was determined by unpaired two‐tailed Student's *t*‐test or one‐way ANOVA followed by Tukey's multiple‐comparisons test, as appropriate.

We next tested the protective effects of MV_normal_ in hydrogen peroxide (H_2_O_2_)‐injured NPCs. H_2_O_2_ stimulation induced a marked decrease in mitochondrial membrane potential, accumulation of reactive oxygen species (ROS), reduced adenosine triphosphate (ATP) production, and disruption of the mitochondrial network, indicating oxidative stress‐induced mitochondrial dysfunction in NPCs. Treatment with MV_normal_ restored mitochondrial membrane potential in a dose‐dependent manner, reduced ROS accumulation, increased ATP levels, and improved the TOMM20‐labelled mitochondrial network (Figures 1d–g and S2a). H_2_O_2_ also increased cGAS, STING, and NLRP3 expression in NPCs, whereas MV_normal_ markedly suppressed activation of this inflammatory axis (Figures 1h and S2b). Because cGAS‐STING‐NLRP3 signaling has been implicated in linking mitochondrial injury to NPC inflammation and disc degeneration, these data suggest that MV_normal_ restrains NPC inflammatory injury by preserving mitochondrial homeostasis [6]. To establish a donor‐cell senescence model, we exposed ADSCs to increasing concentrations of H_2_O_2_ and assessed mitochondrial function, oxidative stress, and senescence‐like changes. Increasing H_2_O_2_ concentrations progressively reduced ADSC mitochondrial membrane potential, increased ROS accumulation, elevated γH2A.X (S139), p‐p53 and p21 expression, enhanced nuclear γH2A.X signals, and increased senescence‐associated β‐galactosidase‐positive area (Figures 1i–m and S2c,d). Moreover, H_2_O_2_ treatment increased the mRNA expression of representative senescence‐associated secretory phenotype (SASP) factors and reduced the proliferative capacity of ADSCs (Figure S2e,f). These results show that H_2_O_2_ treatment induces mitochondrial injury, DNA damage, and senescence‐like phenotypes in ADSCs, providing a donor‐cell model for comparing the functional properties of MV_normal_ and MV_stress_. We then compared the effects of MV_normal_ and MV_stress_ on injured NPCs. PKH26 labelling showed that both vesicle types were taken up by NPCs and entered lysosome‐associated regions (Figure S2g). Functionally, MV_normal_ suppressed H_2_O_2_‐induced cGAS‐STING‐NLRP3 signaling, whereas MV_stress_ maintained cGAS/STING expression and further increased NLRP3 levels (Figures 1n and S2h). In MV_stress_‐treated NPCs, silencing cGAS or STING markedly reduced NLRP3 expression and ROS accumulation, supporting the involvement of the cGAS‐STING pathway (Figure S2i–l). Notably, H_2_O_2_ did not significantly alter cellular mtDNA abundance in donor ADSCs, but increased the levels of ND1, ND2, COX1, and ATP6 in released MVs, indicating enhanced mtDNA loading into MVs (Figure S2m,n). In recipient NPCs, MV_normal_ restored mitochondrial membrane potential, reduced ROS accumulation, and limited mtDNA release from mitochondria, whereas MV_stress_ exerted the opposite effects (Figures 1o–q and S2o–q).

### 2.2 Senescent ADSC‐Derived MVs Fail to Ameliorate Disc Degeneration and Sustain cGAS‐STING‐Mediated Inflammation In Vivo

To determine whether MV_stress_ also loses therapeutic activity in vivo, we established a rat caudal disc puncture model of IVDD and locally injected phosphate‐buffered saline (PBS), MV_normal,_ or MV_stress_ after puncture (Figure 2a). This model reproducibly induces disc height loss, reduced magnetic resonance imaging (MRI) signals, and histological degeneration and is widely used to evaluate IVDD progression and local treatments [7, 23]. Because human ADSC‐derived MVs were administered to rats with an intact immune system, we additionally assessed potential xenogeneic inflammatory responses. Rat ADSCs and their MVs were independently isolated and characterized (Figure S3a–g). Compared with PBS and species‐matched rat ADSC‐derived MVs, human ADSC‐derived MVs did not significantly increase local CD45‐positive cell infiltration or serum IL‐6 and TNF‐α levels, and no obvious histopathological abnormalities were observed in major organs (Figure S3h–k). These findings indicate that, under the single‐dose local administration conditions used here, human ADSC‐derived MVs did not elicit an overt additional inflammatory response attributable to their xenogeneic origin. We next evaluated the effects of the different MV treatments on puncture‐induced disc degeneration. T2‐weighted MRI showed that disc puncture markedly reduced disc signal intensity and increased Pfirrmann grades, confirming successful IVDD induction. MV_normal_ partially preserved T2 signal and significantly reduced Pfirrmann grades, whereas MV_stress_ failed to improve MRI degeneration and maintained high Pfirrmann grades (Figure 2b,c). X‐ray and micro‐computed tomography (micro‐CT) further showed marked narrowing of intervertebral disc space and a reduced disc height index (DHI) in the IVDD group; MV_normal_ partially preserved disc height, whereas MV_stress_ showed poor protection against disc collapse (Figure 2d–f). Histological findings were consistent with imaging results. Hematoxylin and eosin (H&E), Masson, and safranin O‐fast green (SOFG) staining showed typical degeneration in the IVDD group, including loss of nucleus pulposus tissue, disorganized annulus fibrosus, weakened extracellular matrix staining, and global disc structural disruption. MV_normal_ improved nucleus pulposus retention and matrix integrity, whereas the MV_stress_ group still showed severe nucleus pulposus degeneration, annulus fibrosus damage, and matrix loss (Figure 2g–i). Histological scoring further confirmed that MV_normal_ alleviated disc degeneration, whereas MV_stress_ failed to reduce degeneration scores (Figure S3l). We next examined the expression of collagen I, aggrecan, and NLRP3 to assess matrix homeostasis and inflammatory activation. Aggrecan is a core proteoglycan that maintains nucleus pulposus hydration, osmotic pressure, and load‐bearing capacity, and its loss is a major marker of extracellular matrix degeneration in IVDD [24]. Increased collagen I typically reflects fibrotic‐like remodelling of the nucleus pulposus matrix [25]. IVDD increased collagen I and NLRP3 signals and reduced aggrecan expression, indicating matrix imbalance and inflammatory activation. MV_normal_ reduced collagen I and NLRP3 signals and restored aggrecan expression. In contrast, MV_stress_ maintained low aggrecan and high NLRP3 levels, indicating failure to suppress inflammatory matrix degeneration in vivo (Figure 2j,k). Because cGAS‐STING signalling connects cytosolic DNA sensing with inflammatory disc degeneration, we further examined cGAS and STING expression and spatial distribution in disc tissues. Immunofluorescence (IF) revealed enhanced cGAS and STING signals with increased colocalization in the IVDD group. MV_normal_ reduced cGAS/STING signals and weakened their colocalization, whereas the MV_stress_ group retained strong cGAS and STING signals (Figures 2l and S3m). These results demonstrate that MV_normal_ suppresses IVDD‐associated cGAS‐STING inflammation in vivo, whereas MV_stress_ fails to block this inflammatory axis.

**FIGURE 2:**
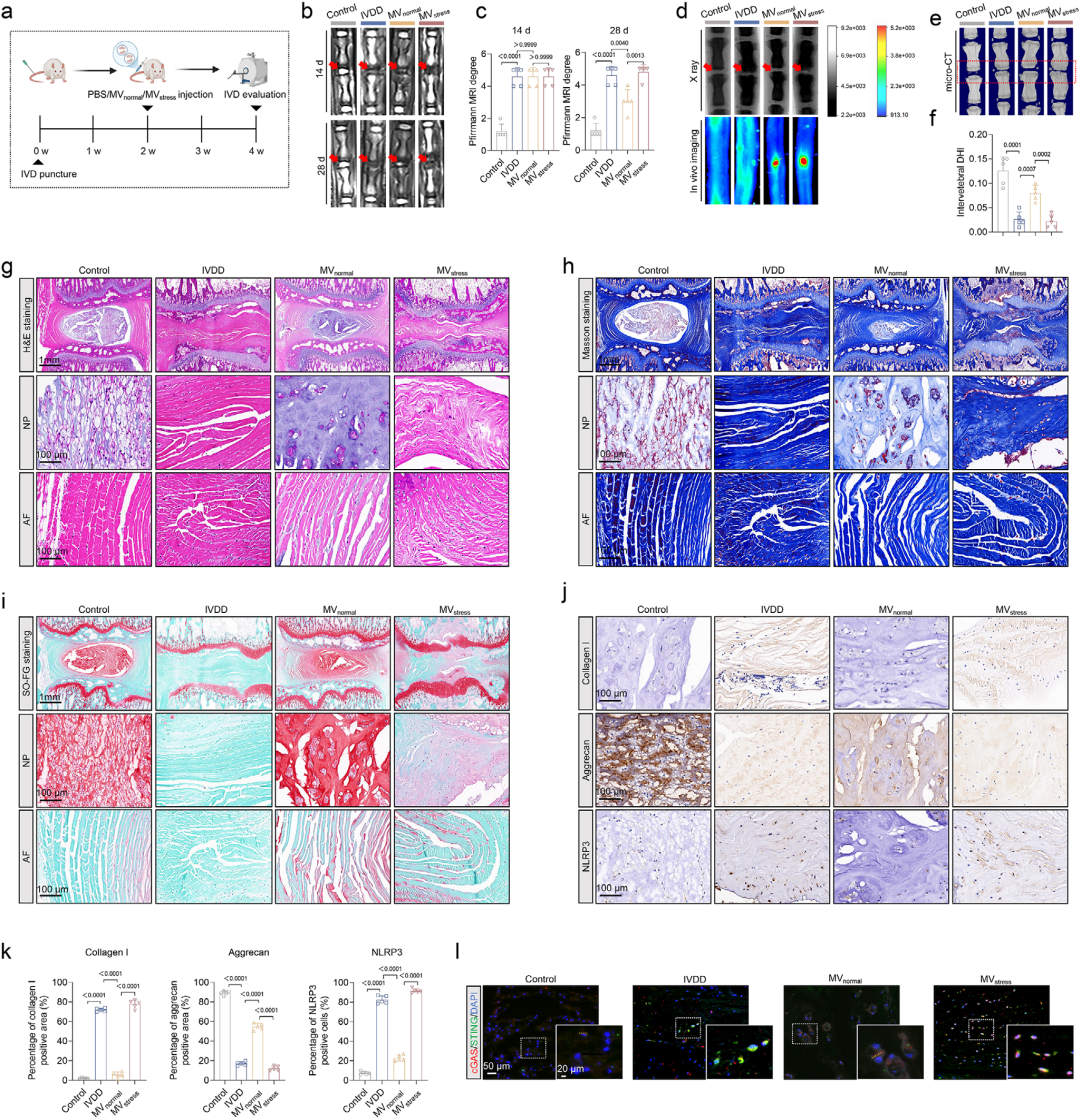
Senescent ADSC‐derived MVs fail to attenuate puncture‐induced IVDD and sustain disc inflammatory degeneration in vivo. (a) Experimental scheme for establishing the rat caudal disc puncture‐induced IVDD model and intradiscal injection of PBS, MV_normal_ or MV_stress_; (b) Representative T2‐weighted MRI images of rat caudal discs at 14 and 28 days after puncture. Red arrows indicate the punctured discs; (c) Pfirrmann grading scores of rat caudal discs at 14 and 28 days after puncture; (d) Representative X‐ray images and in vivo fluorescence imaging of rat caudal discs after intradiscal MV injection. Red arrows indicate the injected discs; (e) Representative micro‐CT reconstruction images of rat caudal discs; (f) Quantification of intervertebral DHI; (g–i) Representative H&E, Masson and SOFG staining images of rat caudal discs from the Control, IVDD, MV_normal_ and MV_stress_ groups. Scale bars, 1 mm and 100 µm; (j) Representative IHC staining images of collagen I, aggrecan and NLRP3 in rat disc tissues. Scale bar, 100 µm; (k) Quantification of collagen I‐positive area, aggrecan‐positive area and NLRP3‐positive cells; (l) Representative IF images of cGAS and STING in rat disc tissues. Scale bars, 50 and 20 µm. Data are presented as mean ± s.d. from n = 5 rats per group, with each rat considered one independent biological replicate. Statistical significance was determined by one‐way or two‐way ANOVA followed by the indicated multiple‐comparisons test, as appropriate.

### 2.3 Senescent ADSCs Preferentially Release Damaged Mitochondria‐Enriched MVs

To define the cargo basis underlying the pro‐inflammatory activity of MV_stress_, we first compared the capacity of ADSC‐derived small extracellular vesicles (sEVs) and MVs to carry mitochondrial components. TEM showed typical membrane‐bound sEVs, and NTA demonstrated a nanoscale size distribution with a median diameter of 135.5 nm and an original particle concentration of 1.2 × 10^11^ particles/mL (Figure 3a,b). These TEM and NTA results confirmed successful isolation of ADSC‐derived sEVs and are consistent with the multi‐parameter characterization recommended for EV analysis [26]. We next used dual labelling with the membrane dye DiI and Mito‐Tracker Green to compare mitochondrial cargo delivery from sEVs and MVs to recipient NPCs (Figure 3c). Flow cytometry showed that Mito‐Tracker Green‐positive signals were markedly higher in NPCs treated with MVs than in those treated with sEVs, indicating stronger mitochondrial cargo transfer by MVs (Figure 3d). IF further confirmed that DiI‐labelled MVs were internalized by NPCs and accompanied by prominent Mito‐Tracker Green signals, whereas mitochondrial signals were weak in the sEV‐treated group (Figure 3e). To independently validate mitochondrial cargo transfer without relying on lipophilic mitochondrial dyes, we transfected donor ADSCs with a genetically encoded mitochondria‐targeted GFP reporter (mito‐GFP). Mito‐GFP colocalized with TOMM20 in donor ADSCs, confirming mitochondrial targeting (Figure S4a). Following vesicle isolation and incubation with recipient NPCs, GFP signals were markedly higher in MV‐treated NPCs than in sEV‐treated NPCs and were associated with DiI‐labelled vesicular structures (Figure S4b). This genetically encoded tracing approach independently confirmed mitochondria‐associated cargo transfer and minimized the possibility that the observed signals resulted from nonspecific dye aggregates. These results indicate that among the vesicle populations secreted by ADSCs, MVs are the predominant carriers of mitochondrial components. To further validate mitochondrial enrichment in MVs, we performed total protein loading control and protein marker analyses of sEVs and MVs. Coomassie brilliant blue staining under equal total protein loading (20 µg per sample) showed distinct protein profiles among cell lysates, sEVs, and MVs, indicating differential cargo composition and providing a loading control for subsequent comparisons of specific proteins between vesicle subtypes (Figure S5a). Ultrathin‐section TEM further demonstrated the presence of mitochondria enclosed within ADSC‐derived MVs (Figure 3f). Western blotting showed enrichment of MV‐associated markers such as Annexin A1 in the MV fraction, together with mitochondrial proteins including HSP60, TOMM20 and VDAC1. These mitochondrial proteins were much weaker in sEVs (Figure 3g,h). Calnexin was not detectably enriched in vesicle fractions, arguing against overt endoplasmic reticulum contamination. Previous studies have reported that MSC‐derived MVs can carry mitochondrial components and regulate recipient‐cell mitochondrial homeostasis, supporting the interpretation that ADSC‐derived MVs function as mitochondrial cargo carriers [2, 13]. After confirming that MVs can carry mitochondrial cargo, we compared the functional state of mitochondrial cargo in MV_normal_ and MV_stress_. Compared with MV_normal_, MV_stress_ showed a lower JC‐1 aggregate/monomer ratio, indicating impaired membrane potential of their mitochondrial cargo. They also showed increased protein carbonyl content and malondialdehyde (MDA) levels, indicating stronger oxidative damage (Figure 3i–k). Thus, senescent ADSCs release MVs that not only contain mitochondrial components but are enriched in damaged and oxidatively stressed mitochondrial cargo. To systematically define cargo remodelling in MV_stress_, we performed tandem mass tag (TMT)‐based quantitative proteomics of MV_normal_ and MV_stress_ (Figure 3l). A total of 4452 proteins were identified. Unsupervised hierarchical clustering of the normalized proteomic profiles showed distinct group‐associated abundance patterns between MV_normal_ and MV_stress_ (Figure S5b). The normalized protein abundance distributions were comparable across the six samples (Figure S5c). Pearson correlation analysis further showed high concordance among biological replicates (Figure S5d), and principal component analysis revealed clear separation between the MV_normal_ and MV_stress_ proteomic profiles (Figure S5e). Using predefined criteria of a fold change ≥1.20 or ≤0.833 together with a Benjamini–Hochberg‐adjusted false discovery rate (FDR) <0.05, 1,370 proteins were identified as differentially abundant, including 746 proteins increased and 624 proteins decreased in MV_stress_. Gene Ontology (GO) enrichment analysis showed significant enrichment of mitochondrial terms, including mitochondrial matrix, mitochondrion, mitochondrial nucleoid, mitochondrial crista junction, mitochondrial inner membrane, and mitochondrial proton‐transporting ATP synthase complex (Figure 3m). GO biological process enrichment further implicated cell motility, cell adhesion, transmembrane transport, aging, protein targeting, signal transduction, cell death, immune system process, and cell cycle, suggesting that cargo remodelling in MV_stress_ extends beyond mitochondrial protein enrichment and may involve broad reprogramming of membrane trafficking, vesicle release, cell adhesion or uptake, senescence and inflammation‐related functions (Figure S5f). Further analysis identified multiple mitochondrial proteins enriched in senescent ADSCs and their MVs. A heatmap showed increased PINK1, VDAC1, TOMM20, TOMM40, ATP5PB and ATP5ME in MV_stress_ (Figure 3n). To determine whether senescence also altered overall MV production, we quantified MV output by NTA using vesicles collected over 24 h from equal numbers of viable donor ADSCs. MV particle output increased from 8.30 ± 0.53 × 10^10^ particles per 10^6^ cells in normal ADSCs to 1.93 ± 0.15 × 10^11^ particles per 10^6^ cells in H_2_O_2_‐induced senescent ADSCs (Figure S5g,h). Consistent with the increased MV output, H_2_O_2_ treatment upregulated MLC1, ROCK1, RhoA, and ARF6, proteins associated with MV biogenesis and membrane trafficking (Figures 3o and S5i). Senescent ADSCs also showed marked changes in mitochondrial stress‐associated proteins, particularly increased PINK1 and VDAC1 (Figures 3p and S5j). Because PINK1 accumulates on the outer mitochondrial membrane after mitochondrial depolarization and serves as a key marker of damaged mitochondrial quality control, its enrichment in senescent ADSCs and their MVs suggested that these MVs may carry PINK1‐marked damaged mitochondrial components [17, 18]. We then directly examined mitochondrial proteins in MV_normal_ and MV_stress_. Under equal total MV protein loading (20 µg per group), Coomassie staining confirmed comparable loading between MV_normal_ and MV_stress_ samples (Figure S5k). Under this condition, MV_stress_ contained markedly higher PINK1, VDAC1, and TOMM20 levels than MV_normal_, whereas calnexin remained undetectable, indicating that mitochondrial protein enrichment was not explained by gross loading differences or organelle contamination (Figures 3q and S5l). As an initial matched‐input comparison, we further adjusted MV samples to comparable Annexin A1 levels, under which PINK1, TOMM20, and VDAC1 remained significantly enriched in MV_stress_ (Figures 3r and S5m). To independently validate this finding using orthogonal normalization strategies, we next compared MV cargo under equal NTA‐defined particle input and per‐donor‐cell normalization. Under equal particle input, PINK1, VDAC1, and TOMM20 remained markedly elevated in MV_stress_, whereas Annexin A1 showed only a modest change (Figure S5n,o). When MVs were normalized to equal numbers of viable donor ADSCs over the same collection period, senescent ADSCs showed increased Annexin A1 output together with substantially greater increases in PINK1, VDAC1 and TOMM20 (Figure S5p–r). Collectively, these results demonstrate that senescence increases both overall MV output and mitochondrial cargo enrichment, and that the elevated mitochondrial protein content of MV_stress_ cannot be explained solely by differences in vesicle abundance or Annexin A1 levels.

**FIGURE 3:**
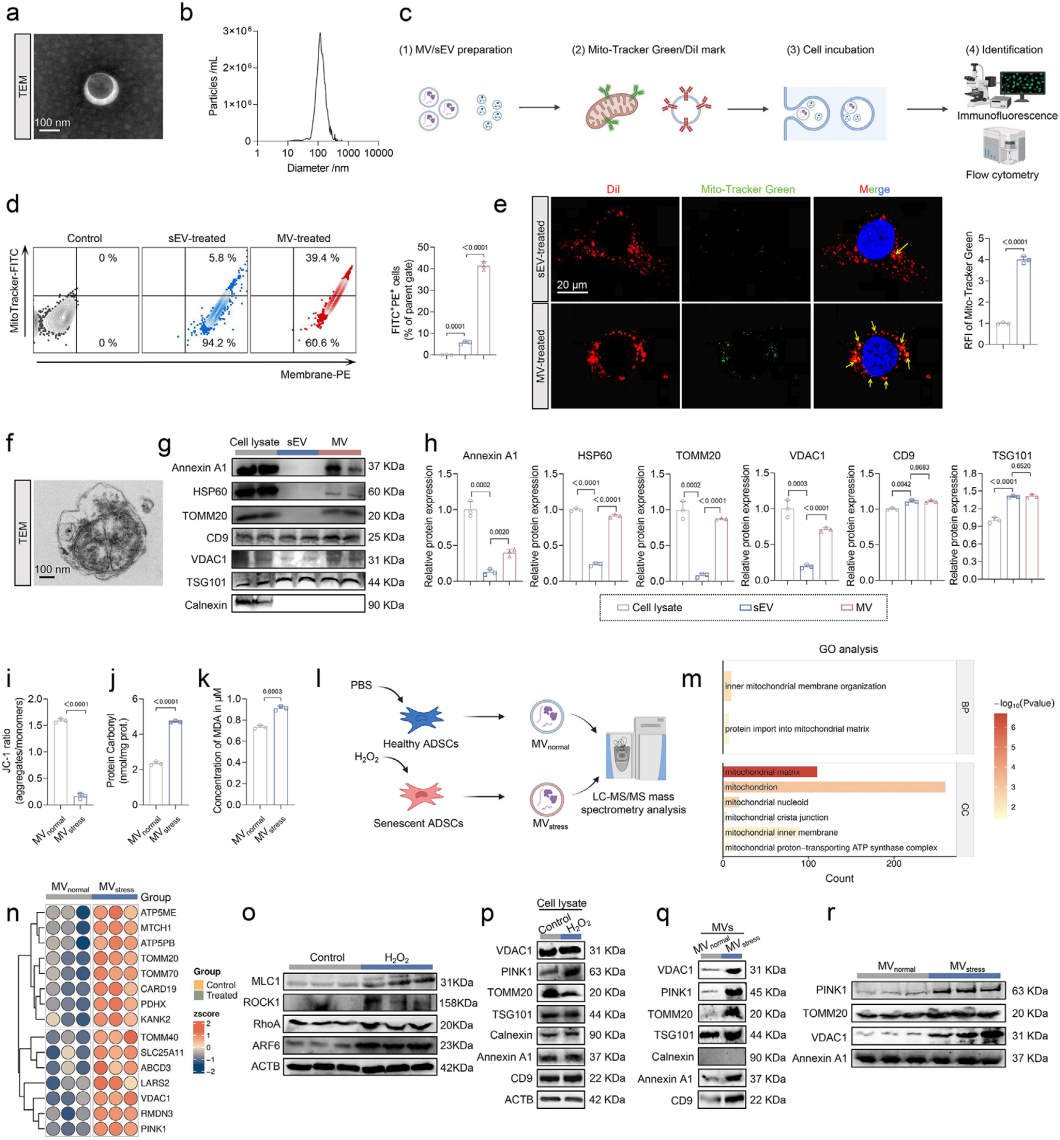
Senescent ADSCs preferentially release damaged mitochondria‐enriched MVs. (a) Representative TEM image of ADSC‐derived sEVs. Scale bar, 100 nm; (b) NTA showing the size distribution of ADSC‐derived sEVs; (c) Schematic illustration of DiI and Mito‐Tracker Green labelling of sEVs or MVs, followed by incubation with recipient NPCs and detection by IF and flow cytometry; (d) Flow cytometry analysis and quantification of vesicle‐delivered Mito‐Tracker Green signals in recipient NPCs; (e) Representative IF images showing DiI‐labelled vesicles and Mito‐Tracker Green signals in recipient NPCs after sEV or MV treatment. Yellow arrows indicate mitochondrial cargo signals associated with internalized MVs; (f) Representative ultrathin‐section TEM image showing membrane‐bound mitochondrial cargo enclosed within ADSC‐derived MVs. Scale bar, 100 nm; (g) WB analysis of EV‐associated markers and mitochondrial proteins in ADSC cell lysates, sEVs and MVs under equal total protein loading; (h) Quantification of protein levels shown in g; (i) JC‐1 aggregate/monomer ratio in MV_normal_ and MV_stress_; (j) Protein carbonyl content in MV_normal_ and MV_stress_; (k) MDA concentration in MV_normal_ and MV_stress_; (l) Schematic illustration of LC‐MS/MS‐based proteomic analysis of MV_normal_ and MV_stress_; (m) GO enrichment analysis of differentially expressed proteins between MV_normal_ and MV_stress_; (n) Heatmap showing selected mitochondrial and mitochondria‐associated proteins identified by proteomic analysis; (o) WB analysis of MLC1, ROCK1, RhoA and ARF6 in ADSCs treated with or without H_2_O_2_; (p) WB analysis of mitochondrial proteins and EV‐associated markers in ADSC cell lysates treated with or without H_2_O_2;_ (q) WB analysis of mitochondrial proteins and EV‐associated markers in MV_normal_ and MV_stress_ under equal total MV protein loading; (r) WB analysis of PINK1, TOMM20 and VDAC1 in MV_normal_ and MV_stress_ under comparable Annexin A1 loading, indicating mitochondrial cargo enrichment per matched MV input. Unless otherwise indicated, quantitative data are presented as mean ± s.d. from n = 3 biological replicates obtained from three independent experiments. For TMT‐based proteomic analysis, n = 3 independently prepared MV samples per group were analyzed. Technical replicates were averaged within each biological replicate. Statistical significance was determined by unpaired two‐tailed Student's *t*‐test or one‐way ANOVA followed by Tukey's multiple‐comparisons test, as appropriate.

### 2.4 HSPA9 Mediates the Sorting of PINK1‐Marked Damaged Mitochondria Into Senescent ADSC‐Derived MVs

Having established the enrichment of damaged mitochondrial cargo in MV_stress_, we next traced the molecular sorting machinery responsible for this process. Proteomic functional screening identified HSPA9 at the intersection between mitochondrion‐related proteins and protein‐trafficking‐related proteins, suggesting that it could connect mitochondrial recognition with vesicular cargo transfer (Figure 4a). In the differential proteomic landscape of MV_normal_ and MV_stress_, HSPA9 was also among the candidate proteins with significant changes (Figure 4b). Western blotting confirmed that H_2_O_2_‐induced ADSC senescence increased intracellular HSPA9 (Figures 4c and S6a). After normalizing MV loading to Annexin A1, HSPA9 was also significantly enriched in MV_stress_ compared with MV_normal_ (Figures 4d and S6b). These findings suggest that HSPA9 may participate in sorting damaged mitochondria into MVs in senescent ADSCs. Subcellular fractionation showed that H_2_O_2_ increased the cytosolic pool of HSPA9, whereas full‐length PINK1 remained predominantly associated with the mitochondrial fraction and accumulated after injury (Figure S6c–e). TOMM20 and ACTB confirmed satisfactory fraction purity. These results support the spatial feasibility of HSPA9 engaging damage‐stabilized PINK1 at the cytosolic surface of damaged mitochondria. To determine whether HSPA9 associates with damaged mitochondria, we performed HSPA9 immunoprecipitation (IP). H_2_O_2_ treatment markedly enhanced the interaction of HSPA9 with VDAC1 and PINK1 (Figure 4e,f). VDAC1 is an outer mitochondrial membrane protein, and PINK1 stably accumulates on the outer mitochondrial membrane after depolarization or damage, serving as a marker of damaged mitochondrial quality control [17, 18]. Thus, enhanced HSPA9‐VDAC1/PINK1 interactions suggest that HSPA9 participates in capturing PINK1‐marked damaged mitochondria. H_2_O_2_ also increased the interaction between HSPA9 and Annexin A1 (Figure 4g,h), indicating that HSPA9 may further link damaged mitochondrial cargo to MV‐associated membrane structures. IF colocalization supported this interpretation. H_2_O_2_ increased colocalization between HSPA9 and PINK1, and line‐scan analysis showed increased overlap of their fluorescence peaks (Figure 4i). Similarly, HSPA9‐VDAC1 colocalization increased after H_2_O_2_ treatment, indicating that HSPA9 is preferentially recruited to PINK1/VDAC1‐marked damaged mitochondrial regions under senescent stress (Figure 4j). Increased HSPA9‐Annexin A1 colocalization further suggested that HSPA9 may connect damaged mitochondria to MV‐associated membrane trafficking structures (Figure 4k). To test the functional requirement for HSPA9 in damaged mitochondrial loading into MVs, we knocked down HSPA9 in ADSCs using siRNA. si‐HSPA9 efficiently reduced HSPA9 expression and suppressed H_2_O_2_‐induced HSPA9 up‐regulation (Figure S6f,g). We then normalized MV loading to Annexin A1 and measured mitochondrial cargo in ADSC‐derived MVs from different treatment groups. H_2_O_2_ increased VDAC1 and PINK1 levels in MVs, whereas HSPA9 knockdown markedly reduced their enrichment (Figures 4l and S6h–j). To independently confirm this effect without relying on Annexin A1 as a loading reference, we further compared MV cargo under equal NTA‐defined particle input and per‐donor‐cell normalization. Under equal particle input, H_2_O_2_ increased VDAC1 and PINK1 levels, whereas HSPA9 knockdown markedly reduced both proteins with only modest changes in Annexin A1 (Figure S6k–n). Under per‐donor‐cell normalization, H_2_O_2_ increased overall MV‐associated Annexin A1 output, while HSPA9 knockdown substantially reduced VDAC1 and PINK1 without a corresponding decrease in Annexin A1 (Figure S6o–r). Together, these complementary normalization approaches indicate that HSPA9 primarily regulates mitochondrial cargo sorting and loading rather than overall MV production. Consistent with the per‐donor‐cell immunoblotting results, ELISA analysis of culture supernatants showed that H_2_O_2_ increased VDAC1 release, whereas HSPA9 knockdown markedly reduced VDAC1 without a corresponding decrease in Annexin A1 (Figure 4m,n), further supporting a selective effect of HSPA9 on mitochondrial cargo release. Finally, HSPA9 knockdown improved the functional quality of MV mitochondrial cargo, as evidenced by an increased JC‐1 aggregate/monomer ratio and reduced protein carbonyl and MDA levels (Figure 4o–q).

**FIGURE 4:**
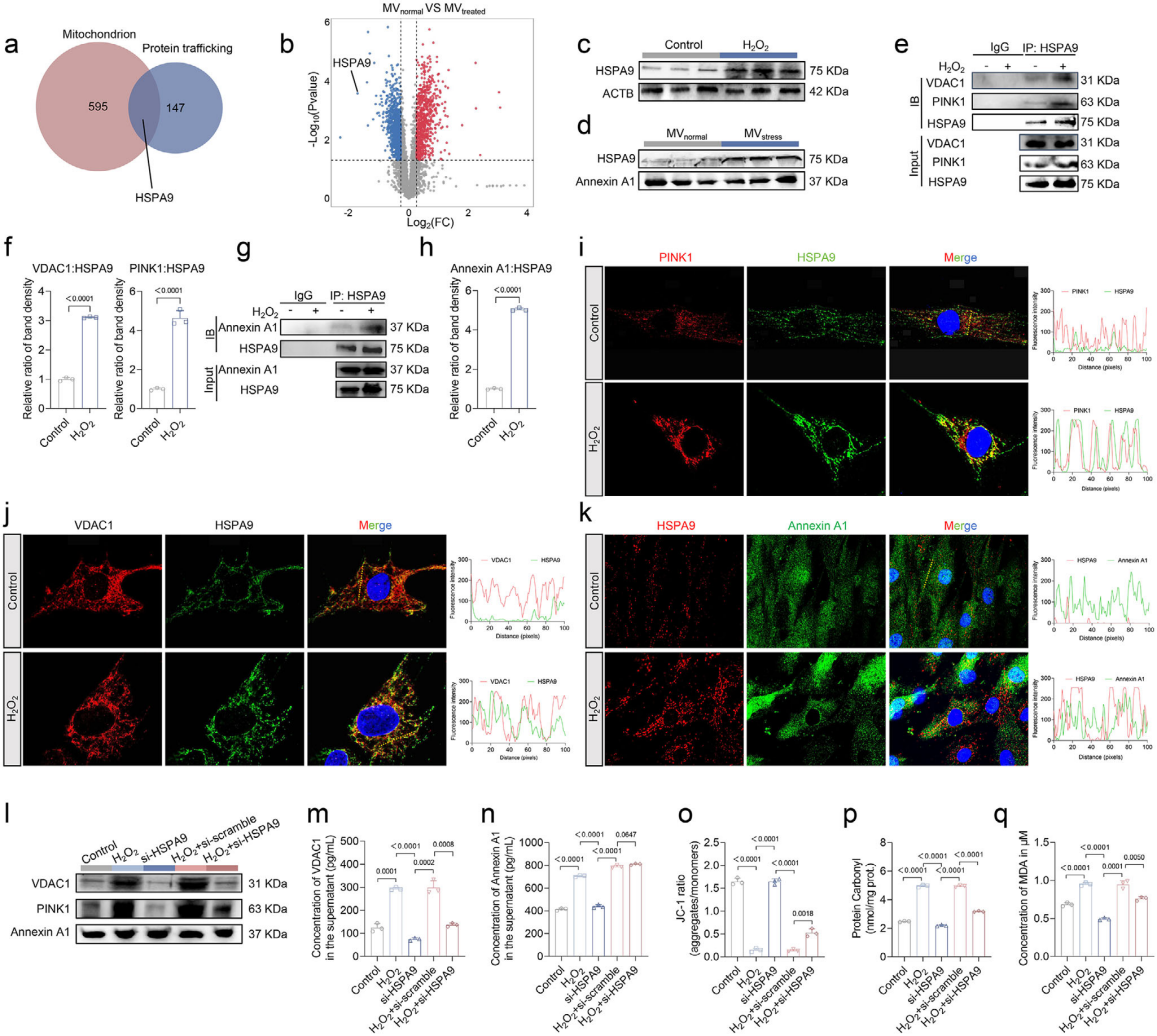
HSPA9 mediates the sorting of PINK1‐marked damaged mitochondria into senescent ADSC‐derived MVs. (a) Venn diagram showing the overlap between mitochondrion‐related proteins and protein‐trafficking‐related proteins identified by proteomic analysis; (b) Volcano plot showing differentially expressed proteins between MV_normal_ and MV_stress_, with HSPA9 indicated; (c) WB analysis of HSPA9 expression in ADSCs treated with or without H_2_O_2_; (d) WB analysis of HSPA9 expression in MV_normal_ and MV_stress_ after normalizing MV loading to Annexin A1; (e) Co‐IP analysis of the interaction between HSPA9 and VDAC1 or PINK1 in ADSCs treated with or without H_2_O_2_; (f) Quantification of VDAC1:HSPA9 and PINK1:HSPA9 interaction levels shown in e; (g) Co‐IP analysis of the interaction between HSPA9 and Annexin A1 in ADSCs treated with or without H_2_O_2_; (h) Quantification of Annexin A1:HSPA9 interaction levels shown in g; (i) Representative IF images and fluorescence intensity profiles showing colocalization of HSPA9 and PINK1 in ADSCs treated with or without H_2_O_2_; (j) Representative IF images and fluorescence intensity profiles showing colocalization of HSPA9 and VDAC1 in ADSCs treated with or without H_2_O_2;_ (k) Representative IF images and fluorescence intensity profiles showing colocalization of HSPA9 and Annexin A1 in ADSCs treated with or without H_2_O_2;_ (l) WB analysis of VDAC1 and PINK1 in ADSC‐derived MV fractions after normalizing MV loading to Annexin A1 under basal or H_2_O_2_‐treated conditions with or without HSPA9 knockdown; (m,n) Concentrations of VDAC1 and Annexin A1 in ADSC culture supernatants collected from equal numbers of donor cells after the indicated treatments; (o) JC‐1 aggregate/monomer ratio in MVs derived from ADSCs after the indicated treatments; (p) Protein carbonyl content in MVs derived from ADSCs after the indicated treatments; (q) MDA concentration in MVs derived from ADSCs after the indicated treatments. Quantitative data are presented as mean ± s.d. from n = 3 biological replicates obtained from three independent experiments. Technical replicates were averaged within each biological replicate and were not treated as independent n values. Statistical significance was determined by unpaired two‐tailed Student's *t*‐test or one‐way ANOVA followed by Tukey's multiple‐comparisons test, as appropriate.

### 2.5 SUMO2/3‐Dependent SUMOylation Potentiates HSPA9‐Mediated Sorting of Damaged Mitochondria into Senescent ADSC‐Derived MVs

After identifying HSPA9 as a mediator of PINK1‐marked damaged mitochondrial loading into MV_stress_, we investigated upstream mechanisms that regulate HSPA9 function. Reactome pathway analysis showed that differentially expressed proteins associated with MV_stress_ were enriched in multiple SUMOylation‐related pathways, including SUMOylation of RNA‐binding proteins, SUMOylation of DNA replication proteins, SUMOylation of ubiquitination proteins, and SUMOylation of DNA damage response and repair proteins (Figures 5a and S7a). Gene set enrichment analysis (GSEA) further showed enrichment of SUMOylation and SUMOylation‐related pathways in senescence‐associated samples (Figures 5b and S7b). These data suggested that SUMOylation may participate in MV cargo remodelling and damaged mitochondrial loading in senescent ADSCs. We next examined global SUMOylation in senescent ADSCs and their MVs. After normalizing MV loading to Annexin A1, both SUMO1‐ and SUMO2/3‐conjugated proteins were higher in MV_stress_ than in MV_normal_ (Figures 5c and S7c,d). Within ADSCs, H_2_O_2_ treatment also increased SUMO1 and SUMO2/3 conjugates, indicating global activation of SUMOylation under senescent stress (Figure 5d). Because HSPA9 was the key mitochondrial cargo‐sorting factor identified above, we next examined whether HSPA9 itself was SUMOylated. HSPA9 IP showed that HSPA9‐associated SUMO2/3 conjugation was much stronger than SUMO1 conjugation, indicating that HSPA9 is primarily associated with SUMO2/3‐related modification (Figure 5e). Reciprocal IP supported this conclusion: SUMO2/3 IP readily enriched HSPA9, whereas HSPA9 signals in SUMO1 immunoprecipitates were weak (Figure 5f,g). Under H_2_O_2_ treatment, HSPA9‐associated SUMO2/3 conjugation increased further, whereas SUMO1‐associated signals remained relatively weak (Figure 5h). These results indicate that senescent stress induces HSPA9 SUMOylation predominantly through SUMO2/3 rather than SUMO1. To further validate HSPA9 SUMO2/3 modification, we reconstituted SUMOylation in 293T cells by expressing GFP‐HSPA9 together with HA‐SUMO2 or HA‐SUMO3 and the SUMO E2 enzyme Myc‐UBC9. HA‐SUMO2 and HA‐SUMO3 both increased HSPA9 SUMOylation signals, and UBC9 further promoted this modification (Figure 5i,j). In contrast, HA‐SUMO1 contributed weakly to HSPA9 modification (Figure 5k). These results are consistent with endogenous IP in ADSCs and support the conclusion that HSPA9 is mainly subject to SUMO2/3‐dependent SUMOylation.

**FIGURE 5:**
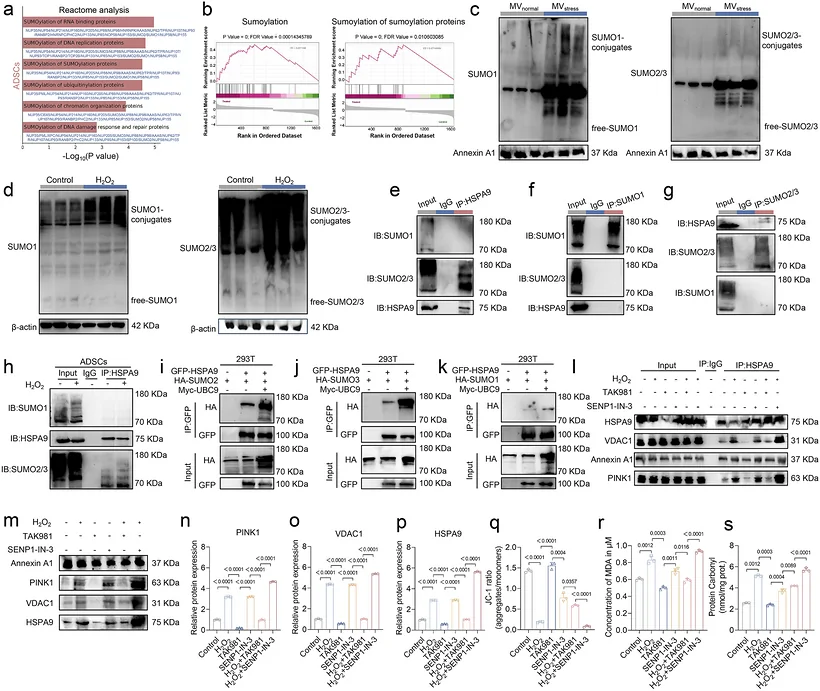
SUMO2/3‐dependent SUMOylation potentiates HSPA9‐mediated sorting of damaged mitochondria into senescent ADSC‐derived MVs. (a) Reactome pathway analysis showing enrichment of SUMOylation‐related pathways in MVs; (b) GSEA plots showing enrichment of SUMOylation and SUMOylation‐related pathways; (c) WB analysis of SUMO1‐ and SUMO2/3‐conjugated proteins in MV_normal_ and MV_stress_. Annexin A1 was used to normalize MV loading; (d) WB analysis of SUMO1‐ and SUMO2/3‐conjugated proteins in ADSCs treated with or without H_2_O_2;_ (e) IP analysis of SUMO1 and SUMO2/3 conjugation associated with HSPA9; (f,g) Reciprocal IP analysis of HSPA9 association with SUMO1 or SUMO2/3 conjugates; (h) IP analysis of HSPA9‐associated SUMO1 and SUMO2/3 conjugation in ADSCs treated with or without H_2_O_2;_ (i–k) IP analysis of GFP‐HSPA9 SUMOylation in 293T cells co‐transfected with HA‐SUMO2, HA‐SUMO3 or HA‐SUMO1, with or without Myc‐UBC9; (l) Co‐IP analysis of HSPA9 interaction with VDAC1, PINK1 and Annexin A1 after pharmacological modulation of SUMOylation using TAK981 or SENP1‐IN‐3 under basal or H_2_O_2_‐treated conditions; (m) WB analysis of Annexin A1, PINK1, VDAC1 and HSPA9 in ADSC‐derived MV fractions after the indicated treatments; (n–p) Quantification of PINK1, VDAC1 and HSPA9 protein expression shown in m; (q) JC‐1 aggregate/monomer ratio in MVs derived from ADSCs after the indicated treatments; (r) MDA concentration in MVs derived from ADSCs after the indicated treatments; (s) Protein carbonyl content in MVs derived from ADSCs after the indicated treatments. Quantitative cell‐based data are presented as mean ± s.d. from n = 3 biological replicates obtained from three independent experiments. Proteomic pathway analyses were based on n = 3 independently prepared MV samples per group. Technical replicates were averaged within each biological replicate. Statistical significance was determined by unpaired two‐tailed Student's *t*‐test or one‐way ANOVA followed by Tukey's multiple‐comparisons test, as appropriate.

We next examined whether global modulation of cellular SUMOylation was associated with changes in HSPA9 binding to damaged mitochondria and MV‐associated structures. To pharmacologically alter the SUMO pathway, we treated ADSCs with TAK981 or SENP1‐IN‐3. TAK981 inhibits the SUMO‐activating enzyme and broadly suppresses SUMO conjugation, whereas SENP1‐IN‐3 inhibits SENP1‐mediated deSUMOylation and promotes the accumulation of SUMO‐conjugated proteins [27, 28, 29]. Consistently, TAK981 reduced H_2_O_2_‐induced accumulation of SUMO2/3 conjugates, whereas SENP1‐IN‐3 further increased SUMO2/3 conjugation (Figure S7e). HSPA9 co‐IP showed that H_2_O_2_ enhanced the interactions of HSPA9 with VDAC1, PINK1, and Annexin A1. TAK981 weakened these interactions, whereas SENP1‐IN‐3 further strengthened them (Figures 5l and S7f). These pharmacological data support a role for the cellular SUMOylation state in modulating HSPA9‐cargo interactions. We then examined MV cargo after SUMOylation modulation. H_2_O_2_ increased HSPA9, PINK1, and VDAC1 levels in ADSC‐derived MVs. TAK981 reduced the enrichment of these proteins in MVs, whereas SENP1‐IN‐3 further increased HSPA9, PINK1, and VDAC1 levels (Figure 5m–p). An Annexin A1‐matched comparison showed a consistent pattern (Figure S7g). To independently validate these findings without relying on Annexin A1 as a loading reference, we further analyzed MV cargo using NTA‐defined particle‐number and per‐donor‐cell normalization. Under equal particle input, H_2_O_2_ increased HSPA9, PINK1, and VDAC1 levels, whereas TAK981 reduced and SENP1‐IN‐3 enhanced their enrichment, with Annexin A1 remaining relatively stable (Figure S7h,i). Under per‐donor‐cell normalization, H_2_O_2_ increased overall Annexin A1 output, while TAK981 markedly reduced H_2_O_2_‐induced mitochondrial cargo release without a corresponding decrease in Annexin A1; conversely, SENP1‐IN‐3 further enhanced HSPA9, PINK1 and VDAC1 output without a parallel increase in Annexin A1 (Figure S7j, k). These complementary normalization approaches indicate that the observed changes in mitochondrial cargo cannot be explained simply by differences in MV abundance or Annexin A1 levels. We next assessed the functional state of MV mitochondrial cargo after SUMOylation modulation. H_2_O_2_ decreased the JC‐1 aggregate/monomer ratio and increased MDA and protein carbonyl levels, indicating greater oxidative injury. TAK981 partially restored the JC‐1 ratio and reduced MDA and protein carbonyl content, whereas SENP1‐IN‐3 further aggravated these injury phenotypes (Figure 5q–s). These pharmacological findings prompted us to further define the HSPA9‐specific SUMOylation mechanism using site‐directed mutagenesis and rescue experiments.

### 2.6 SUMO3 Conjugation at HSPA9 K612 Stabilizes HSPA9 and Licenses Damaged Mitochondrial Cargo Sorting

We next sought to define the specific SUMOylation site on HSPA9 responsible for its damaged mitochondrial cargo‐sorting function. SUMOplot, JASSA, and GPS‐SUMO algorithms were used to predict candidate SUMOylation sites in HSPA9. The intersection of the three prediction outputs identified K612 as the only high‐confidence candidate shared by all algorithms (Figure 6a,b). Structural mapping on an AlphaFold‐predicted HSPA9 model positioned K612 within a relatively accessible region, consistent with a potential SUMO acceptor site (Figures 6c and S8a). Sequence alignment further showed that the K612‐containing region is highly conserved among mammalian HSPA9 orthologues, suggesting an evolutionarily conserved regulatory function (Figure 6d). To test whether K612 mediates HSPA9 SUMOylation, we generated GFP‐HSPA9^WT^ and GFP‐HSPA9^K612R^ and co‐expressed them with HA‐SUMO3 or HA‐SUMO2 in 293T cells. IP showed strong SUMO3 conjugation of HSPA9^WT^, which was markedly reduced by the K612R mutation (Figure 6e). In contrast, under HA‐SUMO2 co‐expression, K612R did not obviously reduce HSPA9‐associated SUMO2 signals (Figure 6f). Thus, K612 is not a generic SUMO2/3 acceptor site but is instead a key lysine required for SUMO3‐dependent HSPA9 modification. Together with the preceding data showing predominant SUMO2/3‐associated HSPA9 signals, these findings focused subsequent analyses on the role of SUMO3‐K612 modification in HSPA9 regulation. We then asked whether K612‐dependent SUMO3 modification controls HSPA9 cargo recognition. After expressing GFP‐HSPA9^WT^ or GFP‐HSPA9^K612R^ in ADSCs, co‐IP showed that K612R markedly reduced HSPA9 binding to PINK1, VDAC1, and Annexin A1 (Figures 6g and S8b). IF colocalization showed concordant results: HSPA9^WT^ displayed clear spatial overlap with PINK1, VDAC1, and Annexin A1, whereas K612R reduced these colocalization signals (Figure 6h–j). These data indicate that SUMO3 modification at K612 affects not only the modification state of HSPA9 but also its ability to connect PINK1‐marked damaged mitochondria with MV‐associated membrane structures. SUMOylation often regulates protein function by altering protein stability, localization, or interaction state [30]. To determine whether K612 modification affects HSPA9 stability, we performed cycloheximide (CHX) chase assays. Under basal conditions, HSPA9^K612R^ decayed faster than HSPA9^WT^; this difference was more pronounced under H_2_O_2_‐induced senescent stress, indicating that K612 is particularly important for maintaining HSPA9 stability under stress (Figure 6k,l). Treatment with the proteasome inhibitor MG132 restored HSPA9^K612R^ levels, indicating that the instability caused by K612R depends primarily on the ubiquitin‐proteasome pathway (Figure 6m,n). To further verify the role of SUMO3 in maintaining HSPA9 stability, we knocked down SUMO3 and examined endogenous HSPA9 protein homeostasis. SUMO3 knockdown reduced HSPA9 protein levels, whereas MG132 partially restored HSPA9 expression, indicating that SUMO3 loss promotes proteasomal degradation of HSPA9 (Figure 6o). HSPA9 IP further showed that SUMO3 knockdown increased HSPA9 ubiquitination, suggesting antagonism between SUMO3 modification and ubiquitin‐dependent degradation of HSPA9 (Figure 6p). CHX chase assays also showed accelerated HSPA9 decay after SUMO3 knockdown, confirming that SUMO3 is required for HSPA9 protein stability (Figure 6q). To directly establish the functional requirement of HSPA9 K612, we performed rescue experiments in HSPA9‐depleted ADSCs by re‐expressing HSPA9^WT^ or HSPA9^K612R^ and collected MVs from the four corresponding donor‐cell conditions (designated MV_rescue1–4_ for H_2_O_2_+si‐scramble+Vector, H_2_O_2_+si‐HSPA9+Vector, H_2_O_2_+si‐HSPA9+HSPA9^WT^ and H_2_O_2_+si‐HSPA9+HSPA9^K612R^, respectively). We first examined the effects of these MVs on recipient NPCs. HSPA9 depletion markedly attenuated cGAS‐STING‐NLRP3 activation, which was restored by MV_rescue3_ derived from HSPA9^WT^‐rescued ADSCs, whereas MV_rescue4_ derived from HSPA9^K612R^‐rescued ADSCs showed a substantially weaker rescue effect (Figure S8c,d). We next assessed mitochondrial cargo loading into MVs. HSPA9 knockdown markedly reduced PINK1 and VDAC1 levels in MVs; this reduction was restored by re‐expression of HSPA9^WT^ but only partially rescued by HSPA9^K612R^, despite comparable HSPA9 expression levels (Figure S8e,f). Together, these data identify K612 SUMOylation as a functional determinant that stabilizes HSPA9 by limiting ubiquitin‐proteasome‐mediated degradation and supports HSPA9‐dependent mitochondrial cargo sorting and downstream pro‐inflammatory activity (Figure 6r).

**FIGURE 6:**
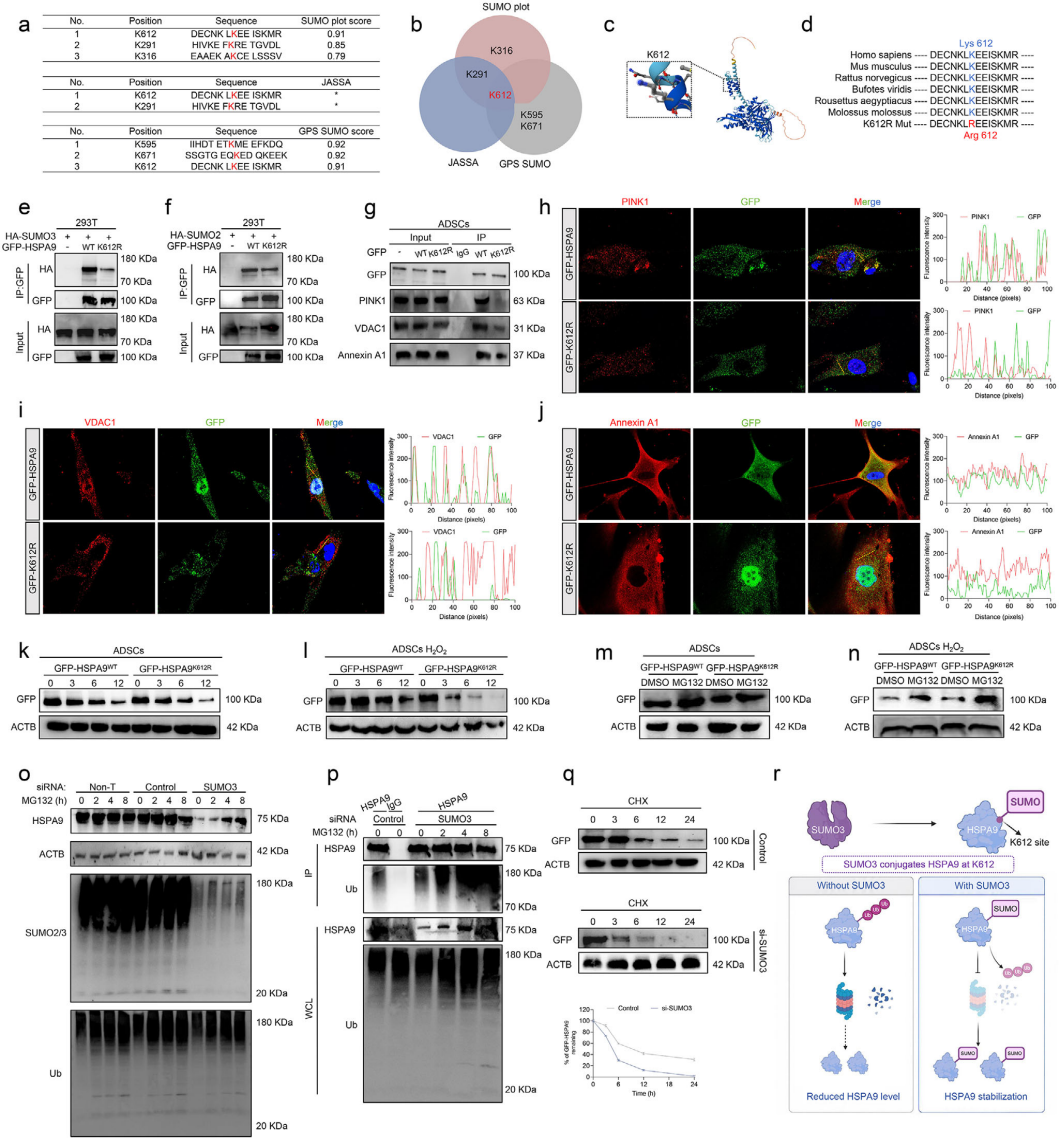
SUMO3 conjugation at HSPA9 K612 stabilizes HSPA9 and licenses damaged mitochondrial cargo sorting. (a) Prediction of candidate SUMOylation sites in HSPA9 using SUMOplot, JASSA and GPS‐SUMO algorithms; (b) Venn diagram showing the overlap of predicted SUMOylation sites identified by the three algorithms. K612 was identified as the shared candidate site; (c) Structural mapping of K612 in the AlphaFold‐predicted HSPA9 model. The enlarged view shows the spatial location of K612; (d) Sequence alignment showing evolutionary conservation of the K612‐containing region across species and schematic indication of the K612R mutation; (e) IP analysis showing SUMO3 conjugation of GFP‐HSPA9^WT^ and its reduction in the GFP‐HSPA9^K612R^ mutant in 293T cells co‐transfected with HA‐SUMO3; (f) IP analysis showing SUMO2‐associated GFP‐HSPA9 signals in 293T cells co‐transfected with HA‐SUMO2 and GFP‐HSPA9^WT^ or GFP‐HSPA9^K612R^; (g) Co‐IP analysis of the interaction between GFP‐HSPA9 and PINK1, VDAC1 or Annexin A1 in ADSCs expressing GFP‐HSPA9^WT^ or GFP‐HSPA9^K612R^; (h–j) Representative IF images and fluorescence intensity profiles showing colocalization of GFP‐HSPA9^WT^ or GFP‐HSPA9^K612R^ with PINK1, VDAC1 or Annexin A1 in ADSCs; (k,l) CHX chase analysis of GFP‐HSPA9^WT^ and GFP‐HSPA9^K612R^ stability in ADSCs under basal or H_2_O_2_‐treated conditions; (m,n) WB analysis of GFP‐HSPA9^WT^ and GFP‐HSPA9^K612R^ in ADSCs treated with DMSO or MG132 under basal or H_2_O_2_‐treated conditions; (o) WB analysis of HSPA9, SUMO2/3 conjugates and ubiquitinated proteins in ADSCs transfected with control siRNA or si‐SUMO3 and treated with MG132 for the indicated durations; (p) IP analysis of HSPA9 ubiquitination in ADSCs transfected with control siRNA or si‐SUMO3 and treated with MG132; (q) CHX chase analysis of GFP‐HSPA9 stability in ADSCs transfected with control siRNA or si‐SUMO3; (r) Schematic model showing that SUMO3 conjugation at HSPA9 K612 stabilizes HSPA9 by limiting ubiquitin‐proteasome‐mediated degradation. Quantitative cell‐based data are presented as mean ± s.d. from n = 3 biological replicates obtained from three independent experiments. Technical replicates were averaged within each biological replicate. Statistical significance was determined by unpaired two‐tailed Student's *t*‐test, one‐way ANOVA, or two‐way ANOVA followed by the appropriate multiple‐comparisons test.

### 2.7 Activated PINK1 Directly Engages HSPA9 to License Damaged Mitochondrial Cargo Sorting

The preceding data positioned HSPA9 as a central adaptor for damaged mitochondrial loading into MVs in senescent ADSCs. A key remaining question was how HSPA9 recognizes PINK1‐marked damaged mitochondria and converts this mitochondrial quality‐control signal into a vesicular cargo‐sorting event. To define the structural basis of this interaction, we first generated domain‐deletion and functional mutants of HSPA9 and PINK1. HSPA9 contains an N‐terminal ATPase domain and a C‐terminal PBD, whereas PINK1 contains a mitochondrial targeting sequence (MTS), a transmembrane domain (TMD), a KD, and a C‐terminal domain (CTD) [31, 32] (Figure 7a,b). Domain‐mapping co‐IP showed that deletion of the PINK1 region containing the KD markedly reduced its interaction with HSPA9. A kinase‐inactive PINK1 mutant carrying K219A/D362A/D384A substitutions also showed substantially impaired HSPA9 binding. Conversely, carbonyl cyanide m‐chlorophenyl hydrazone (CCCP)‐induced mitochondrial depolarization strongly enhanced the PINK1‐HSPA9 interaction (Figure 7c). These results indicate that PINK1 KD integrity and stress‐induced activation state are key determinants of HSPA9 recognition. We then mapped the PINK1‐binding region within HSPA9. Co‐IP using HSPA9 truncation mutants showed that the HSPA9 PBD is particularly important for PINK1 binding. CCCP enhanced the interaction between PINK1 and HSPA9^WT^, whereas PBD deletion markedly weakened this binding (Figure 7d). Thus, HSPA9 primarily uses its PBD to recognize stress‐activated PINK1, suggesting that the HSPA9‐PINK1 interaction forms a direct recognition module for damaged mitochondrial entry into the MV loading route. To rationalize this interaction structurally, we built an HSPA9‐PINK1 complex using an AlphaFold3‐predicted model and performed a 100 ns all‐atom molecular dynamics simulation (Figures 7e and S9a,b). The AlphaFold3 model showed a tightly interlocked interface between HSPA9 and PINK1, providing a reasonable starting structure for dynamic refinement. During simulation, the overall root mean square deviation (RMSD) reached a stable plateau after early conformational relaxation, with an average RMSD of approximately 5.56 Å and a major distribution peak of approximately 5.54 Å. Although single‐chain RMSD values for HSPA9 and PINK1 were approximately 4.50 and 5.64 Å, respectively, the interface RMSD remained low, with an average of approximately 2.33 Å and a major peak of approximately 2.57 Å, indicating that the binding interface remained stable in a dynamic environment (Figures 7f and S9c–f). Root mean square fluctuation (RMSF) analysis showed that high‐flexibility regions were concentrated in peripheral loops and terminal regions, whereas interface residues exhibited low fluctuations, indicating a stable local contact network (Figures 7g and S9g). Radius of gyration (Rg) analysis showed compact folding of HSPA9 and PINK1 throughout the simulation, with average Rg values of approximately 33.28 and 30.46 Å, respectively. Secondary‐structure evolution and Ramachandran analysis further supported reasonable backbone conformations without gross structural destabilization (Figures 7h and S9h–m). PCA and free‐energy landscape analysis showed that the complex gradually converged from the initial conformation toward a low‐free‐energy ensemble, supporting a stable binding tendency (Figures 7i and S9n–q). Interface analysis revealed pronounced electrostatic complementarity between HSPA9 and PINK1 at the binding region (Figure 7j). Non‐covalent interaction statistics showed that the interface maintained a high number of total contacts, with an average of approximately 86.71 interactions. Hydrophobic contacts and hydrogen bonds contributed most prominently, with averages of approximately 49.40 and 33.79, respectively, while salt bridges and pi‐cation interactions provided smaller but persistent auxiliary contributions (Figure S10a–e). Residue‐level interaction maps identified several high‐frequency nodes, including Tyr404, Arg312 and Glu231 on PINK1 and Asp151 on HSPA9 (Figure S10f,g). The two‐dimensional interaction map showed that the HSPA9‐PINK1 interface is stabilized by aromatic/hydrophobic anchoring, multiple hydrogen bonds, and charge locks (Figure 7k). Molecular mechanics/generalized Born surface area (MM‐GBSA) analysis showed a strongly negative binding free energy, with an average ΔG_total of approximately −52.79 kcal/mol, and residue‐level hotspot contributions matched the high‐frequency interface nodes, providing thermodynamic support for complex stability (Figure S10h‐j). To experimentally validate the predicted direct interaction, we expressed and purified recombinant human HSPA9 and PINK1 proteins. The protein expression report indicated that HSPA9 (amino acids 1–679) and PINK1 (amino acids 111–581) were expressed in Escherichia coli with N‐His‐SUMO and C‐His tags and purified for in vitro binding assays (Figure S11a). We then used surface plasmon resonance (SPR) to measure direct binding. HSPA9 was immobilized on a CM5 chip by amine coupling at approximately 3000 response units (RU), and PINK1 was injected as the analyte at 0, 62.5, 125, 250, 500, and 1000 nM in a multi‐cycle kinetic assay. After subtraction of reference channel and zero‐concentration background signals, sensorgrams showed concentration‐dependent binding of PINK1 to immobilized HSPA9 (Figure 7l). Kinetic fitting yielded an association rate constant (Ka) of 3.728 × 10^4^ (1/Ms) and a dissociation rate constant (Kd) of 0.01753 (1/s), corresponding to an equilibrium dissociation constant (KD) of 4.704 × 10^−7^ M. Steady‐state fitting yielded a KD of 6.459 × 10^−7^ M. Both fitting approaches demonstrated submicromolar affinity between HSPA9 and PINK1, supporting direct binding between the purified proteins (Figures 7l and S11b). To exclude reliance on a single in vitro detection platform, we further validated the HSPA9‐PINK1 interaction using a split‐ubiquitin yeast two‐hybrid system. The HSPA9‐PINK1 pair supported growth on a stringent selection medium, whereas the negative control did not, indicating a stable interaction in an independent protein‐interaction system (Figure 7m). Together with domain mapping, molecular dynamics simulation, and SPR, these data establish a multi‐layered evidence chain for direct HSPA9‐PINK1 engagement. Finally, we knocked down PINK1 to test its requirement for HSPA9‐mediated mitochondrial cargo sorting. To compare cargo composition under equal MV input, Annexin A1 was used as an MV loading control to normalize MV loading across groups. Under comparable Annexin A1 levels, PINK1 knockdown reduced PINK1 signals in MVs and weakened H_2_O_2_‐induced HSPA9 and VDAC1 enrichment (Figures 7n and S11c). To independently confirm this effect, we further analyzed MV cargo using NTA‐based and per‐donor‐cell normalization. Under equal particle input, PINK1 knockdown markedly reduced PINK1, HSPA9, and VDAC1 levels in MVs under both basal and H_2_O_2_‐treated conditions, whereas Annexin A1 showed only minor changes (Figure S11d,e). Under per‐donor‐cell normalization, H_2_O_2_ increased overall MV‐associated Annexin A1 output, while PINK1 knockdown substantially reduced PINK1, HSPA9, and VDAC1 without a corresponding decrease in Annexin A1 (Figure S11f,g). Co‐IP further showed that H_2_O_2_ increased HSPA9 binding to PINK1, VDAC1, and Annexin A1, whereas PINK1 knockdown reduced HSPA9 interactions with VDAC1 and Annexin A1 (Figure S11h–k). These findings identify stress‐activated PINK1 as a mitochondrial recognition platform that engages HSPA9 and supports selective loading of damaged mitochondrial cargo into MVs.

**FIGURE 7:**
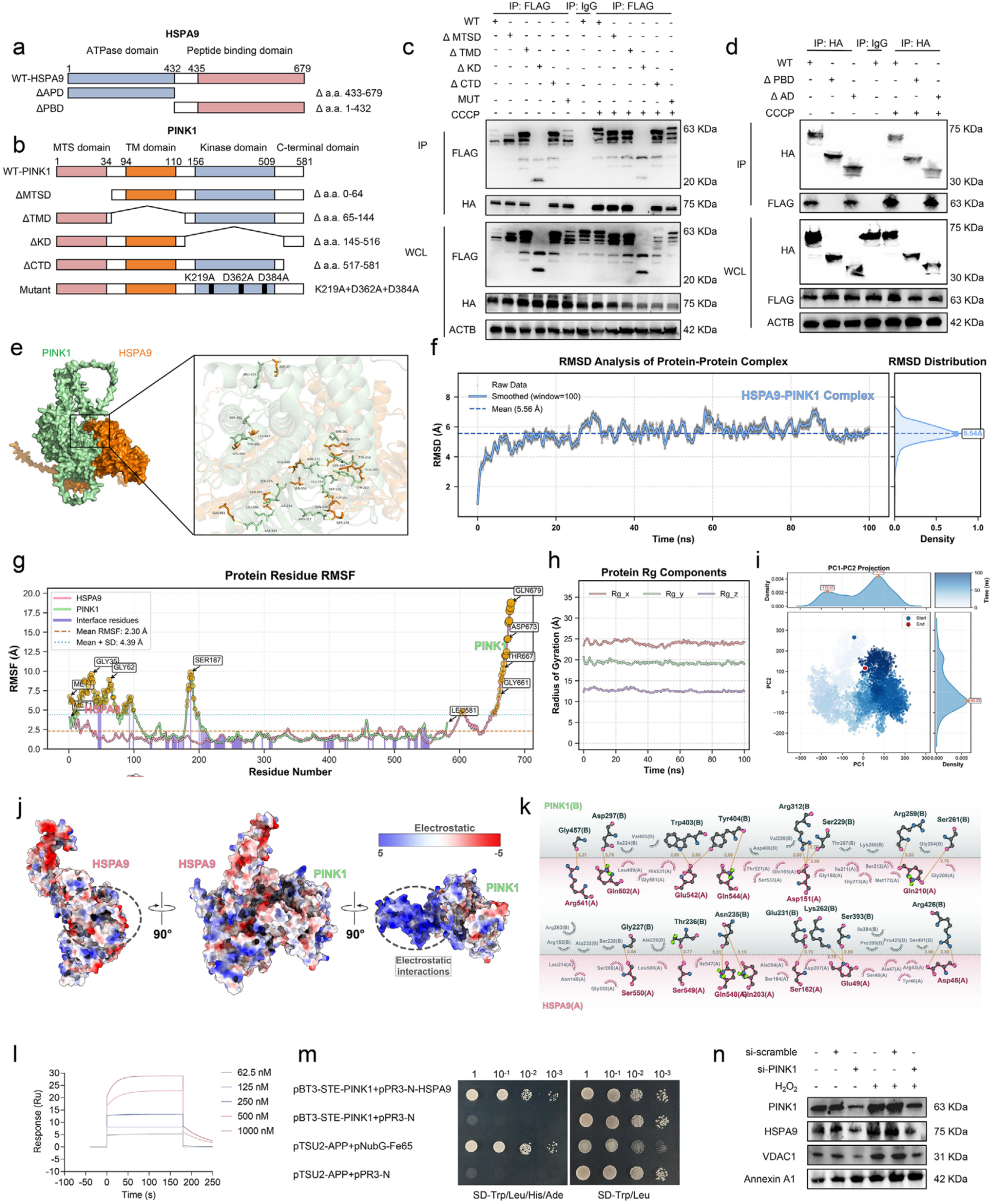
Activated PINK1 directly engages HSPA9 to license damaged mitochondrial cargo sorting. (a) Schematic representation of HSPA9 domain organization and truncation mutants. HSPA9 contains an N‐terminal ATPase domain and a C‐terminal peptide‐binding domain (PBD); (b) Schematic representation of PINK1 domain organization and deletion or kinase‐inactive mutants. PINK1 contains a mitochondrial targeting sequence (MTS), transmembrane domain (TMD), kinase domain (KD) and C‐terminal domain (CTD). The kinase‐inactive mutant contains K219A, D362A and D384A substitutions; (c) Co‐IP analysis of HA‐HSPA9 interaction with FLAG‐tagged PINK1 WT or PINK1 mutants under basal or CCCP‐treated conditions; (d) Co‐IP analysis of FLAG‐PINK1 interaction with HA‐tagged HSPA9 WT or HSPA9 truncation mutants under basal or CCCP‐treated conditions; (e) AlphaFold3‐predicted structural model of the HSPA9–PINK1 complex followed by molecular dynamics refinement, with a magnified view of the predicted binding interface; (f) RMSD analysis of the HSPA9–PINK1 complex during 100 ns molecular dynamics simulation; (g) RMSF analysis of HSPA9 and PINK1 residues, with interface residues indicated; (h) Radius of gyration components of the HSPA9–PINK1 complex during simulation; (i) Principal component analysis of the HSPA9–PINK1 simulation trajectory; (j) Electrostatic potential surface analysis of the HSPA9–PINK1 interface; (k) Two‐dimensional interaction map showing key interfacial contacts between HSPA9 and PINK1 in the energy‐minimized conformation; (l) SPR sensorgrams showing concentration‐dependent binding of purified PINK1 to immobilized HSPA9; (m) Split‐ubiquitin yeast two‐hybrid assay validating the HSPA9–PINK1 interaction; (n) WB analysis of PINK1, HSPA9 and VDAC1 in ADSC‐derived MV fractions after PINK1 knockdown under basal or H_2_O_2_‐treated conditions, with Annexin A1 used as an MV loading control. Cell‐based experiments were performed with n = 3 biological replicates from three independent experiments, and representative results are shown. Computational analyses, SPR, and split‐ubiquitin yeast two‐hybrid assays were analyzed as described in the Methods and were not treated as biological replicates.

### 2.8 Mitophagy Counterbalances HSPA9‐Mediated Export of Damaged Mitochondria in Senescent ADSCs

PINK1‐marked damaged mitochondria are classically cleared intracellularly through PINK1‐Parkin‐mediated mitophagy [17, 18]. Because we found that HSPA9 promotes entry of PINK1‐marked damaged mitochondria into MVs, we asked how this route relates to mitophagy. We first knocked down HSPA9 in ADSCs and examined PINK1‐Parkin mitophagy‐associated proteins. Under H_2_O_2_‐induced senescent stress, HSPA9 silencing markedly altered the expression pattern of mitochondrial quality‐control proteins, including PINK1, TOMM20, SQSTM1, Parkin, and the LC3II/LC3I ratio, with further accumulation of PINK1 and Parkin, reduced TOMM20, and increased LC3II/LC3I (Figures 8a and S12a). These findings suggest that when HSPA9‐mediated MV export of damaged mitochondria is blocked, PINK1‐marked mitochondria are redirected toward intracellular mitophagy‐associated processing. Thus, HSPA9‐mediated MV export and PINK1‐Parkin mitophagy may represent two parallel fates for damaged mitochondrial quality control. To modulate donor‐cell mitophagy, we treated H_2_O_2_‐induced senescent ADSCs with 3‐methyladenine (3‐MA), rapamycin (RAPA), or NMN. 3‐MA inhibits autophagy initiation, whereas RAPA promotes autophagy by inhibiting mechanistic target of rapamycin (mTOR). NMN, an NAD^+^ precursor, has been reported to restore mitochondrial homeostasis and autophagy/mitophagy activity, including DCT‐1/NIX‐, ULK1‐, and PINK1‐Parkin‐associated mitophagy, through NAD^+^ replenishment [33, 34, 35]. We therefore used NMN as a metabolic intervention to improve mitochondrial quality control in senescent ADSCs. IF showed that H_2_O_2_ increased TOMM20‐LC3 colocalization, indicating recruitment of damaged mitochondria to autophagic structures. 3‐MA reduced this colocalization, whereas RAPA and NMN further enhanced TOMM20‐LC3 colocalization (Figures 8b and S12b). Western blotting showed that 3‐MA, RAPA, and NMN remodelled PINK1, TOMM20, SQSTM1, Parkin, and LC3II/LC3I expression patterns, indicating effective modulation of mitochondrial quality‐control state in ADSCs (Figures 8c and S12c). Mitochondrial functional assays supported these findings. H_2_O_2_ reduced mitochondrial membrane potential and ATP levels in ADSCs and induced ROS accumulation. 3‐MA further aggravated mitochondrial dysfunction, whereas RAPA and NMN partially restored mitochondrial membrane potential and ATP production and reduced ROS levels (Figures 8d–f and S12d,e). Thus, promoting mitophagy or improving mitochondrial homeostasis alleviates mitochondrial damage in senescent ADSCs, whereas autophagy inhibition aggravates damaged mitochondrial accumulation. In particular, NMN improved mitochondrial membrane potential, ATP production, and ROS levels and increased TOMM20‐LC3 colocalization, suggesting that NAD^+^ replenishment shifts mitochondrial quality control in senescent ADSCs away from damaged mitochondrial export and back toward intracellular clearance. We then examined how MVs generated under different donor‐cell treatment conditions affected mitochondrial status in recipient NPCs. Compared with the H_2_O_2_‐injured condition, MV_stress_ failed to restore NPC mitochondrial structure, and MVs from 3‐MA‐treated ADSCs retained strong mitochondrial injury effects. In contrast, MVs from RAPA‐ or NMN‐treated ADSCs improved TOMM20/DNA signal distribution and increased mitochondrial colocalization metrics in NPCs (Figure 8g,h). These results indicate that the mitophagy state of donor ADSCs determines the mitochondrial impact of their MVs on recipient NPCs. To further determine how donor‐cell mitophagy influences HSPA9‐mediated damaged mitochondrial loading into MVs, we examined HSPA9 interactions with PINK1, VDAC1, and Annexin A1. Co‐IP showed that H_2_O_2_ markedly enhanced HSPA9 binding to PINK1, VDAC1, and Annexin A1. 3‐MA further increased these interactions, whereas RAPA and NMN reduced them (Figure 8i,j). Thus, when mitophagy is impaired, HSPA9 is more likely to connect PINK1/VDAC1‐marked damaged mitochondria to MV‐associated membrane structures. Conversely, mitophagy activation or restoration of mitochondrial homeostasis reduces HSPA9‐mediated damaged mitochondrial loading. An Annexin A1‐matched comparison showed that H_2_O_2_ increased HSPA9, VDAC1, and PINK1 levels in MVs, with further enrichment after 3‐MA treatment and reduced levels after RAPA or NMN treatment (Figure 8k,l). To independently validate these changes, we further analyzed MV cargo using NTA‐based and per‐donor‐cell normalization. Under equal particle input, the same pattern of HSPA9, VDAC1, and PINK1 enrichment was retained, whereas Annexin A1 showed comparatively smaller changes (Figure S12f,g). Per‐donor‐cell normalization similarly showed enhanced mitochondrial cargo output after H_2_O_2_ and 3‐MA treatment, whereas RAPA and NMN reduced HSPA9, VDAC1, and PINK1 output (Figure S12h,i). Consistently, MVs from 3‐MA‐treated ADSCs showed higher MDA and protein carbonyl levels and a lower JC‐1 aggregate/monomer ratio. In contrast, RAPA and NMN reduced oxidative injury markers and improved mitochondrial membrane potential status in MVs (Figure 8m–o). Together, these findings reveal a mitochondrial quality‐control fate switch in senescent ADSCs, whereby PINK1‐marked damaged mitochondria are dynamically partitioned between PINK1‐Parkin‐mediated mitophagic clearance and HSPA9‐mediated loading into MVs; when intracellular mitophagy becomes insufficient, this balance shifts toward extracellular export, thereby converting unresolved mitochondrial damage into a transmissible pro‐inflammatory signal.

**FIGURE 8:**
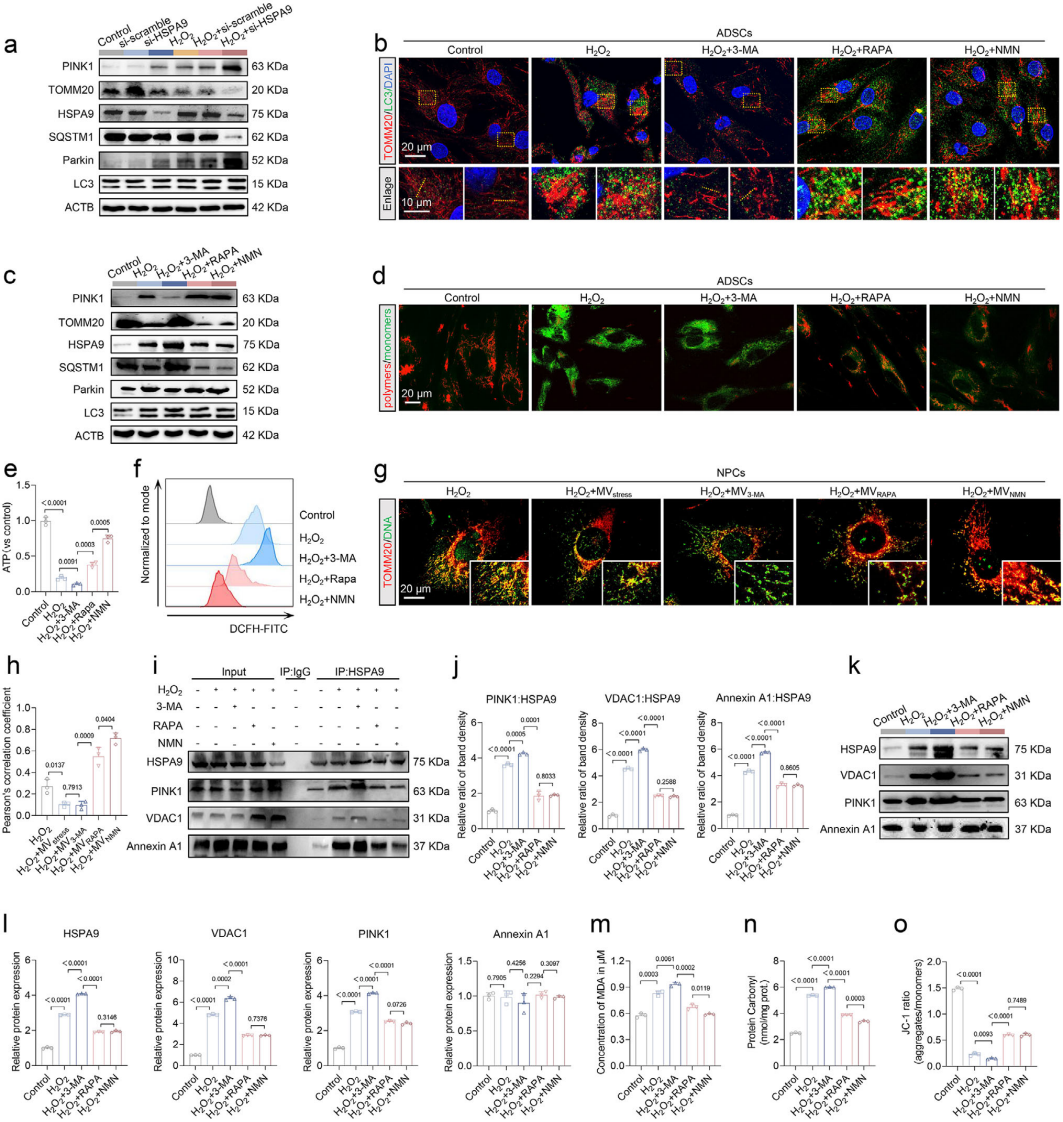
Mitophagy counterbalances HSPA9‐mediated export of damaged mitochondria in senescent ADSCs. (a) WB analysis of PINK1‐Parkin mitophagy‐associated proteins in ADSCs after HSPA9 knockdown under basal or H_2_O_2_‐treated conditions; (b) Representative IF images showing TOMM20 and LC3 colocalization in ADSCs treated with H_2_O_2_, 3‐MA, rapamycin (RAPA) or NMN. Scale bars, 20 µm and 10 µm; (c) WB analysis of PINK1, TOMM20, HSPA9, SQSTM1, Parkin and LC3 in ADSCs treated as indicated; (d) Representative JC‐1 fluorescence images showing mitochondrial membrane potential in ADSCs after the indicated treatments. Scale bar, 20 µm; (e) ATP content in ADSCs after the indicated treatments; (f) DCFH flow cytometry analysis of ROS accumulation in ADSCs after the indicated treatments; (g) Representative IF images showing TOMM20 and DNA in H_2_O_2_‐injured NPCs treated with MVs derived from senescent ADSCs exposed to 3‐MA, RAPA or NMN. Scale bar, 20 µm; (h) Quantification of TOMM20‐DNA colocalization in NPCs shown in g; (i) Co‐IP analysis of HSPA9 interaction with PINK1, VDAC1 and Annexin A1 in ADSCs after pharmacological modulation of mitophagy; (j) Quantification of PINK1:HSPA9, VDAC1:HSPA9 and Annexin A1:HSPA9 interaction levels shown in i; (k,l) WB analysis and quantification of HSPA9, VDAC1 and PINK1 in ADSC‐derived MV fractions after the indicated treatments, with Annexin A1 used as an MV loading control; (m) MDA concentration in MVs derived from ADSCs after the indicated treatments; (n) Protein carbonyl content in MVs derived from ADSCs after the indicated treatments; (o) JC‐1 aggregate/monomer ratio in MVs derived from ADSCs after the indicated treatments. Quantitative data are presented as mean ± s.d. from n = 3 biological replicates obtained from three independent experiments. Technical replicates were averaged within each biological replicate and were not treated as independent n values. Statistical significance was determined by one‐way or two‐way ANOVA followed by the appropriate multiple‐comparisons test.

### 2.9 Donor‐Cell Reprogramming Restores the Therapeutic Efficacy of Senescent ADSC‐Derived MVs

The above findings suggested that the pathological activity of MV_stress_ is not determined simply by its senescent origin, but arises from donor‐cell mitochondrial quality‐control imbalance and HSPA9‐dependent loading of damaged mitochondrial cargo into MVs. Based on this mechanism, we attempted to remodel MV cargo at the donor‐cell level by using NMN to restore mitochondrial homeostasis in senescent ADSCs and si‐HSPA9 to block damaged mitochondrial sorting into MVs. We first assessed the effects of NMN and HSPA9 silencing on mitochondrial status in H_2_O_2_‐induced senescent ADSCs. JC‐1 flow cytometry showed that H_2_O_2_ markedly reduced mitochondrial membrane potential, whereas NMN restored the JC‐1 aggregate/monomer ratio. si‐HSPA9 alone did not effectively improve mitochondrial membrane potential, but combined NMN and si‐HSPA9 maintained a high mitochondrial membrane potential (Figures 9a,b and S13a). Consistently, H_2_O_2_ induced ROS accumulation and increased protein carbonyl and MDA levels. NMN reduced these oxidative injury markers, and combined NMN and si‐HSPA9 also effectively suppressed ROS, protein carbonylation, and lipid peroxidation (Figures 9c,d and S13b,c). These results indicate that NMN primarily restores mitochondrial functional status in senescent ADSCs, whereas HSPA9 silencing blocks the HSPA9‐dependent sorting route by which damaged mitochondrial cargo enters MVs. We therefore defined the MVs generated from senescent ADSCs treated with NMN plus si‐HSPA9 as modified MVs (MV_modified_).

**FIGURE 9:**
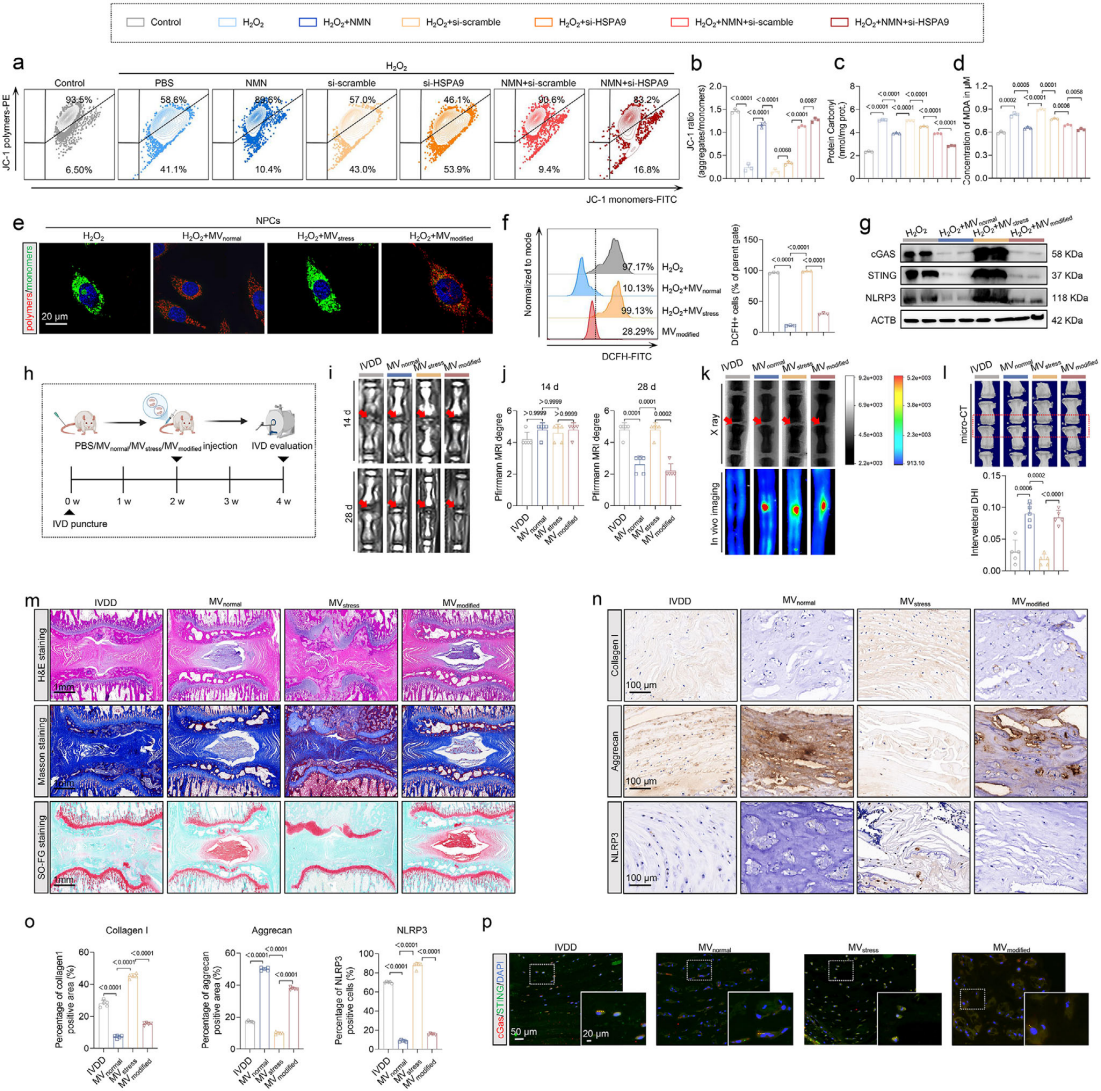
Donor‐cell reprogramming restores the therapeutic efficacy of senescent ADSC‐derived MVs. (a) JC‐1 flow cytometry analysis of mitochondrial membrane potential in ADSCs treated with H_2_O_2_, NMN, si‐scramble, si‐HSPA9 or combined NMN and si‐HSPA9; (b) Quantification of the JC‐1 aggregate/monomer ratio shown in a; (c) Protein carbonyl content in ADSCs after the indicated treatments; (d) MDA concentration in ADSCs after the indicated treatments; (e) Representative JC‐1 fluorescence images showing mitochondrial membrane potential in H_2_O_2_‐injured NPCs treated with MV_normal_, MV_stress_ or MV_modified_. Scale bar, 20 µm; (f) DCFH flow cytometry analysis and quantification of ROS levels in H_2_O_2_‐injured NPCs treated with the indicated MVs; (g) WB analysis of cGAS, STING and NLRP3 in H_2_O_2_‐injured NPCs treated with MV_normal_, MV_stress_ or MV_modified_; (h) Experimental scheme for intradiscal injection of PBS, MV_normal,_ MV_stress_ or MV_modified_ in the rat puncture‐induced IVDD model; (i) Representative T2‐weighted MRI images of rat caudal discs at 14 and 28 days after puncture and MV treatment. Red arrows indicate the punctured discs; (j) Pfirrmann grading scores of rat caudal discs at 14 and 28 days after puncture; (k) Representative X‐ray images and in vivo fluorescence imaging of rat caudal discs after intradiscal MV injection. Red arrows indicate the injected discs; (l) Representative micro‐CT images and quantification of intervertebral DHI; (m) Representative H&E, Masson and SOFG staining images of rat caudal discs from the IVDD, MV_normal_, MV_stress_ or MV_modified_ groups. Scale bars, 1 mm and 100 µm; (n) Representative immunohistochemical staining images of collagen I, aggrecan and NLRP3 in rat disc tissues. Scale bar, 100 µm; (o) Quantification of collagen I‐positive area, aggrecan‐positive area and NLRP3‐positive cells; (p) Representative IF images of cGAS and STING in rat disc tissues. Scale bars, 50 µm and 20 µm. For in vitro experiments, quantitative data are presented as mean ± s.d. from n = 3 biological replicates obtained from three independent experiments. For in vivo experiments, n = 5 rats per group, with each rat considered one independent biological replicate. Technical replicates were averaged within each biological replicate. Statistical significance was determined by one‐way or two‐way ANOVA followed by the appropriate multiple‐comparisons test.

We next tested the function of MV_modified_ in H_2_O_2_‐injured NPCs. Compared with MV_stress_, MV_modified_ significantly improved mitochondrial membrane potential in NPCs, reduced DCFH fluorescence, and suppressed ROS accumulation (Figure 9e,f). Western blotting and quantification further showed that MV_stress_ enhanced cGAS, STING, and NLRP3 expression in NPCs, whereas MV_modified_ effectively suppressed this inflammatory axis, with a trend resembling MV_normal_ (Figures 9g and S13d). These results demonstrate that donor‐cell treatment with NMN+si‐HSPA9 remodels the functional identity of MV_stress_, shifting it away from a pro‐inflammatory phenotype and restoring its mitochondrial protective and anti‐inflammatory activity. To determine whether MV_modified_ restores therapeutic efficacy in vivo, we injected PBS, MVnormal, MV_stress,_ or MV_modified_ into rat caudal discs after puncture‐induced IVDD (Figure 9h). MRI showed that the IVDD and MV_stress_ groups had reduced T2 signal and high Pfirrmann grades, whereas MV_normal_ and MV_modified_ preserved T2 signal and reduced Pfirrmann grades (Figure 9i,j). X‐ray, in vivo fluorescence imaging, and micro‐CT further showed that MV_modified_ alleviated IVDD‐associated disc space narrowing and DHI loss, whereas MV_stress_ remained poorly protective against disc height collapse (Figure 9k,l). Histological staining supported the imaging findings. H&E, Masson, and SOFG staining showed marked nucleus pulposus loss, annulus fibrosus disorganization, and reduced matrix staining in the IVDD and MV_stress_ groups. In contrast, MV_normal_ and MV_modified_ improved disc architecture and matrix preservation (Figure 9m). Histological scoring further showed that MV_modified_ significantly reduced disc degeneration (Figure S13e). Immunohistochemistry (IHC) showed that MV_modified_ reduced collagen I and NLRP3 signals and restored aggrecan expression, indicating attenuation of fibrotic‐like matrix remodelling, matrix loss, and inflammatory activation in IVDD (Figure 9n,o). IF and line‐scan analysis further showed that MVmodified reduced cGAS and STING signals in disc tissues, whereas MV_stress_ maintained strong cGAS/STING activation (Figures 9p and S13f). Thus, restoring donor‐cell mitochondrial homeostasis while limiting HSPA9‐dependent cargo sorting can recover the therapeutic activity of MV_stress_.

## 3 Discussion

This study establishes donor‐cell mitochondrial quality control as a determinant of the functional identity of ADSC‐derived MVs during IVDD. Donor‐cell senescence not only increased MV output but also remodeled MV composition toward PINK1‐marked, oxidatively damaged mitochondrial cargo. After transfer to recipient NPCs, these MVs impaired mitochondrial homeostasis and activated cGAS‐STING‐NLRP3 signaling, whereas MV_normal_ preserved mitochondrial function and restrained inflammation. Previous studies have shown that cellular senescence alters EV abundance, molecular composition, and biological activity [15], while cytosolic escape of mtDNA can activate the cGAS‐STING‐NLRP3 axis and promote inflammatory degeneration in NPCs [6]. Our findings connect these previously separate processes by showing that senescent donor cells can package mitochondrial danger signals into MVs and transmit them to recipient cells. Thus, ADSC‐derived MVs do not possess an invariant therapeutic identity; their biological activity is determined by donor‐cell state and, more specifically, by the quality of the mitochondrial cargo they carry.

These findings should be considered in the context of emerging evidence that extracellular mitochondrial export constitutes an alternative arm of mitochondrial quality control. Rabas et al. demonstrated that PINK1 promotes the production of mtDNA‐containing EVs and enables mtDNA‐dependent signaling in recipient tumor cells [36]. Howard et al. subsequently described a Parkin‐independent form of secretory mitophagy in which EVs released under metabolic stress were enriched in PINK1, TOM20, VDAC1, and other mitochondrial fission‐ and mitophagy‐associated proteins [37]. Todkar et al. further showed that OPA1/SNX9‐dependent mitochondrial‐derived vesicles direct mitochondrial proteins toward EV secretion, whereas Parkin diverts damaged mitochondrial material toward lysosomal degradation and limits the extracellular release of mitochondrial DAMPs [2]. In another physiological context, energetically stressed adipocytes released oxidatively damaged but respiration‐competent mitochondrial particles in EVs, eliciting an adaptive mitohormetic response in the heart [1]. Together, these studies demonstrate that mitochondrial material can enter extracellular vesicular routes and that its biological consequence depends on cargo quality and recipient‐cell context. The advance of the present study is therefore not the observation of mitochondrial EV export itself, but the identification of a senescence‐responsive sorting mechanism that determines mitochondrial cargo fate. Specifically, we define a SUMO3‐HSPA9‐PINK1 module that redirects PINK1‐marked damaged mitochondrial material toward MV loading and links this process to cGAS‐STING‐NLRP3 activation and degenerative tissue injury.

Mechanistically, HSPA9 emerged as the molecular adaptor connecting damaged mitochondria with MV‐associated membrane structures. Subcellular fractionation showed that senescent stress increased the cytosolic pool of HSPA9, whereas full‐length PINK1 remained predominantly associated with damaged mitochondria, providing spatial access for HSPA9 to engage PINK1 at or near the cytosolic mitochondrial surface. Domain mapping and direct‐binding assays further indicated that the HSPA9 peptide‐binding domain recognizes the kinase‐containing region of stress‐activated PINK1. This interaction was controlled by SUMO3 conjugation at HSPA9 K612. SUMO3 modification stabilized HSPA9 by limiting its ubiquitination and proteasome‐dependent degradation, thereby sustaining its interactions with PINK1, VDAC1, and Annexin A1. Importantly, re‐expression of HSPA9^WT^ in HSPA9‐depleted ADSCs restored mitochondrial cargo loading into MVs and the inflammatory activity of these MVs in recipient NPCs, whereas HSPA9^K612R^ produced only a partial rescue. These data position K612 SUMOylation as a functional licensing event rather than merely a biochemical modification. The SUMO3‐HSPA9‐PINK1 axis consequently integrates stress sensing, adaptor stabilization, damaged‐mitochondrial recognition, and vesicular cargo selection within a single molecular framework.

This sorting mechanism also places MV export in direct competition with canonical PINK1‐Parkin mitophagy. PINK1 accumulation on depolarized mitochondria and subsequent Parkin activation normally initiate ubiquitin‐dependent clearance of damaged mitochondria [17, 18]. In the present study, blocking HSPA9‐dependent export shifted mitochondrial quality‐control markers toward enhanced intracellular mitophagy‐associated processing. Conversely, autophagy inhibition intensified HSPA9‐cargo interactions and increased the loading of damaged mitochondrial components into MVs, whereas RAPA and NMN reduced mitochondrial cargo loading and improved cargo quality. NAD^+^ replenishment has previously been shown to restore mitochondrial homeostasis by enhancing mitophagy in aging‐related settings [33], consistent with the effects of NMN observed here. These results support a model in which intracellular mitophagy and extracellular MV export represent counterbalancing fates for PINK1‐marked mitochondria. When intracellular clearance is insufficient, HSPA9‐mediated export may serve as an alternative disposal route; however, this process transfers the burden of damaged mitochondrial material from donor cells to recipient tissues. This cargo‐fate model also provides a therapeutic rationale for targeting the donor cell. Combining NMN‐mediated restoration of mitochondrial homeostasis with HSPA9 silencing reduced pathological cargo incorporation and shifted MV_stress_ from an inflammatory toward a protective phenotype, thereby restoring its therapeutic efficacy in vivo.

Several limitations should be acknowledged. First, H_2_O_2_‐induced senescence reproduces oxidative stress, DNA damage, SASP induction, and mitochondrial dysfunction but does not fully recapitulate chronological aging. Validation in naturally aged human ADSCs and additional senescence models will be required to establish the generality of this mechanism. Second, increased MV‐associated mtDNA and cGAS/STING loss‐of‐function experiments support mitochondrial DAMP transfer as a driver of recipient‐cell inflammation, but selective depletion of vesicular mtDNA would help distinguish the contribution of mtDNA from that of oxidized mitochondrial proteins or other mitochondrial cargo species. Third, although particle‐number and per‐donor‐cell normalization support selective mitochondrial cargo enrichment, the MV‐enriched fractions obtained by differential centrifugation remain heterogeneous, and higher‐resolution separation approaches may further define the responsible vesicle subpopulation. The pharmacological modulators used to alter SUMOylation and mitophagy also have broader cellular effects, although the K612R rescue experiments and genetic perturbation of HSPA9 provide more specific mechanistic support. Finally, the absence of overt local or systemic inflammation after a single xenogeneic MV administration does not exclude immune responses following repeated or long‐term dosing. All in vivo experiments were performed in male rats, and potential sex‐dependent differences in MV responses remain to be evaluated. Collectively, this study defines a SUMO3‐HSPA9‐PINK1 cargo‐sorting axis that links donor‐cell senescence to the extracellular transmission of damaged mitochondrial material and identifies mitochondrial cargo fate as a checkpoint determining whether ADSC‐derived MVs promote tissue repair or inflammatory degeneration.

## 4 Materials and Methods

### 4.1 Ethics Statement

Human nucleus pulposus tissues were obtained from six patients undergoing clinically indicated spinal surgery for idiopathic scoliosis, and human adipose tissues were obtained from six adult donors undergoing elective cosmetic thigh liposuction (Table S1 and Table S2). Written informed consent was obtained from all adult participants before tissue collection. For participants younger than 18 years, written informed consent was obtained from a parent or legal guardian, with age‐appropriate assent obtained where applicable. The collection and experimental use of human tissues were approved by the Ethics Committee of Union Hospital, Tongji Medical College, Huazhong University of Science and Technology (approval No. 0448 and S347). All animal procedures were approved by the Institutional Animal Care and Use Committee of Union Hospital, Tongji Medical College, Huazhong University of Science and Technology (approval No. 4571) and were performed in accordance with institutional guidelines and the Guide for the Care and Use of Laboratory Animals. All studies involving human specimens and animals were conducted in accordance with relevant ethical regulations. The animal experiments are reported in accordance with the ARRIVE 2.0 guidelines.

### 4.2 Isolation and Culture of Human and Rat ADSCs

ADSCs were isolated from adipose tissues using protocols adapted from published stem‐cell and IVDD studies [6, 7, 12, 38]. Briefly, adipose tissues were washed three times with PBS (Gibco, 10010023) containing 1% penicillin‐streptomycin (Gibco, 15140122), minced into small fragments, and digested with 0.1% collagenase type I (Sigma‐Aldrich, C0130) at 37°C with gentle agitation. The digested suspension was filtered through a 40 µm cell strainer (Corning, 352340) and centrifuged at 800 rpm for 10 min. The stromal vascular fraction was resuspended and cultured in Dulbecco's modified Eagle's medium/F12 (DMEM/F12; Gibco, 11330032) supplemented with 10% fetal bovine serum (FBS; Gibco, 10099141) and 1% penicillin‐streptomycin at 37°C in a humidified incubator with 5% CO_2_. Cells at passages 3 to 6 were used for experiments. Rat ADSCs were isolated from Sprague‐Dawley (SD) rat adipose tissues using the same procedure and culture conditions and were used for phenotypic characterization and preparation of rat ADSC‐derived MVs for xenogeneic immune‐response assessment.

### 4.3 Phenotypic Characterization of ADSCs

For flow cytometric phenotyping, ADSCs were detached using 0.25% trypsin‐EDTA (Gibco, 25200056), washed with PBS, and incubated with fluorophore‐conjugated antibodies against CD73‐PE (BioLegend, 344004), CD90‐FITC (BioLegend, 328108), CD105‐FITC (BioLegend, 323204), or HLA‐DR‐PE (BioLegend, 307606) for 30 min at 4°C. Cells were washed and analyzed using a flow cytometer (BD Biosciences, FACSCanto II). For osteogenic differentiation, ADSCs were cultured in osteogenic induction medium (Cyagen, HUXMA‐90021), followed by Alizarin Red S staining (Solarbio, G1452). For adipogenic differentiation, ADSCs were cultured in adipogenic induction medium (Cyagen, HUXMA‐90031), followed by Oil Red O staining (Solarbio, G1262).

### 4.4 Isolation and Culture of Human Primary NPCs

Human primary NPCs were isolated from nucleus pulposus tissues obtained from patients undergoing spinal surgery. All human tissue collection and use were approved by the Ethics Committee of Union Hospital, Tongji Medical College, Huazhong University of Science and Technology, and written informed consent was obtained from all donors before sample collection. The degeneration grade of each intervertebral disc was evaluated preoperatively by MRI according to the Pfirrmann grading system. Nucleus pulposus tissues were collected intraoperatively under sterile conditions and immediately transported to the laboratory in sterile PBS (Servicebio, G4202) supplemented with 1% penicillin‐streptomycin (Gibco, 15140122). For primary NPC isolation, nucleus pulposus tissues were carefully separated from annulus fibrosus and cartilaginous endplate remnants under a stereomicroscope. The tissues were washed three times with cold PBS to remove blood and debris, minced into approximately 1 mm^3^ fragments, and digested with 0.4% collagenase type II (Gibco, 17101015) in serum‐free DMEM/F‐12 (Gibco, 11330032) for 4 h at 37°C with gentle agitation. The digested suspension was filtered through a 40‐µm cell strainer (Falcon, 352340) and centrifuged at 800 rpm for 5 min. The cell pellet was washed twice with PBS and resuspended in complete NPC culture medium consisting of DMEM/F12 supplemented with 15% fetal bovine serum (FBS; Gibco, 10099141C) and 1% penicillin‐streptomycin. For cryopreservation, human primary NPCs were resuspended in serum‐free, protein‐free rapid cell freezing medium (Shanghai Life‐iLab Biotech Co., Ltd., AC05L033) and stored in liquid nitrogen until use. For experiments, cryopreserved NPCs were rapidly thawed at 37°C, washed to remove residual cryopreservation medium, seeded in culture dishes, and maintained at 37°C in a humidified incubator containing 5% CO_2_. The culture medium was replaced every 2–3 days. When cells reached approximately 80–90% confluence, they were passaged using 0.25% trypsin–EDTA (Gibco, 25200056). Passage 2 human primary NPCs were used for all in vitro experiments to minimize phenotypic drift during culture. For oxidative stress experiments, NPCs were treated with H_2_O_2_ (Sigma‐Aldrich, H1009) at 200 µM for 24 h. For MV treatment experiments, H_2_O_2_‐injured NPCs were incubated with ADSC‐derived MVs as described in the corresponding experimental design.

### 4.5 Enzyme‐Linked Immunosorbent Assay

The concentrations of VDAC1 and Annexin A1 in ADSC culture supernatants were measured using enzyme‐linked immunosorbent assay (ELISA) kits from Boster Biological Technology Co., Ltd. (Boster/Bosterbio, Wuhan, China) according to the manufacturer's instructions. Briefly, culture supernatants were collected from equal numbers of donor ADSCs after the indicated treatments and centrifuged to remove cell debris. Samples and standards were added to ELISA plates and incubated according to the kit protocols. After washing, detection antibody, enzyme conjugate, and substrate solution were sequentially added. The reaction was stopped with stop solution, and absorbance was measured at 450 nm using a microplate reader. Concentrations were calculated from standard curves and normalized where appropriate.

### 4.6 Cell Treatments

ADSC senescence was induced by treating cells with H_2_O_2_ (Sigma–Aldrich, H1009) at 100 µM for 48 h. NPC injury was induced with 200 µM H_2_O_2_ for 24 h. For mitochondrial quality‐control experiments, ADSCs were treated with 5 mM 3‐MA (Selleck, S2767), 100 nM RAPA (Selleck, S1039), or 500 µM NMN (MedChemExpress, HY‐F0004) for 24 h in the presence of H_2_O_2_, unless otherwise indicated. For generation of modified MVs, senescent ADSCs were treated with NMN during H_2_O_2_‐induced senescence and transfected with si‐HSPA9 before MV collection. For PINK1 activation, cells were treated with 10 µM CCCP (Sigma‐Aldrich, C2759) for 6 h. SUMOylation was pharmacologically modulated using 1 µM TAK981 (MedChemExpress, HY‐111789) or 10 µM SENP1‐IN‐3 (MedChemExpress, HY‐134596) for 24 h. Proteasome inhibition was performed using 10 µM MG132 (Selleck Chemicals, S2619, Selleckchem.com) for 6 h, unless a time‐course treatment was specifically indicated. Protein synthesis was blocked with 50 µg/mL CHX (MedChemExpress, HY‐12320), and cells were collected at the indicated time points for protein stability assays.

### 4.7 siRNA Transfection and Plasmids

siRNAs targeting HSPA9, PINK1, or SUMO3, together with the corresponding negative control siRNA, were custom‐synthesized by Corues Biotechnology (Nanjing, China). The siRNA sequences are listed in Table S3. ADSCs were transfected with siRNAs using Lipofectamine 3000 Transfection Reagent (Invitrogen, L3000015) in Opti‐MEM Reduced Serum Medium (Gibco, 31985070) according to the manufacturer's instructions. Unless otherwise indicated, siRNAs were used at a final concentration of 50 nM, and cells were harvested 48–72 h after transfection for subsequent experiments. GFP‐tagged HSPA9 wild‐type and K612R mutant plasmids, HA‐tagged SUMO1, SUMO2 and SUMO3 plasmids, Myc‐UBC9 plasmid, FLAG‐tagged PINK1 wild‐type and mutant constructs, HA‐tagged HSPA9 truncation constructs and the corresponding empty vectors were generated by gene synthesis or site‐directed mutagenesis by HySigen Biosciences (Suzhou, China). All plasmid constructs were verified by Sanger sequencing before use. For plasmid transfection, ADSCs or HEK293T cells were transfected with the indicated plasmids using Lipofectamine 3000 Transfection Reagent (Invitrogen, L3000015) in Opti‐MEM Reduced Serum Medium (Gibco, 31985070) according to the manufacturer's protocol, and cells were collected 48–72 h after transfection for immunoblotting, IP, or IF analyses.

### 4.8 Quantitative Real‐Time PCR and mtDNA Quantification

Total RNA was extracted from ADSCs using the FastPure Cell/Tissue Total RNA Isolation Kit V2 (Vazyme Biotech Co., Ltd., Nanjing, China; RC112‐01) according to the manufacturer's instructions. RNA concentration and purity were assessed before reverse transcription. Equal amounts of total RNA were reverse‐transcribed into cDNA using HiScript III RT SuperMix for qPCR (+gDNA wiper) (Vazyme Biotech Co., Ltd.; R323‐01). Quantitative real‐time PCR (qRT‐PCR) was performed using ChamQ Universal SYBR qPCR Master Mix (Vazyme Biotech Co., Ltd.; Q711‐02) according to the manufacturer's instructions. Relative mRNA expression was calculated using the 2^−ΔΔCt^ method and normalized to GAPDH. Primer sequences for IL6, CXCL8, CCL2, ACTB and GAPDH are provided in Table S4.

For mtDNA quantification, total DNA was extracted from ADSCs or purified MV preparations using the FastPure Blood/Cell/Tissue/Bacteria DNA Isolation Mini Kit (Vazyme Biotech Co., Ltd.; DC112‐01) according to the manufacturer's instructions. For MV‐associated mtDNA measurements, equal NTA‐defined particle numbers were used as input for DNA extraction, and the abundance of each mitochondrial gene was expressed relative to the corresponding control group. mtDNA abundance was quantified by qPCR using ChamQ Universal SYBR qPCR Master Mix (Vazyme Biotech Co., Ltd.; Q711‐02) with primers targeting the mitochondrial genes ND1, ND2, COX1, and ATP6. For cellular samples, mtDNA abundance was normalized to the nuclear β‐globin gene and expressed as the relative mtDNA‐to‐nuclear DNA ratio. Primer sequences for β‐globin, ND1, ND2, COX1 and ATP6 are provided in Table S5. Unless otherwise indicated, experiments were performed with three independent biological replicates. Each biological sample was analyzed in technical triplicate, and technical replicate Ct values were averaged before statistical analysis.

### 4.9 EDU Proliferation Assay

Cell proliferation was assessed using the SF594 EdU Cell Proliferation Assay Kit (Red Fluorescence) (Beijing Solarbio Science & Technology Co., Ltd., Beijing, China; Cat. No. CA1174) according to the manufacturer's instructions. ADSCs were seeded in culture plates and subjected to the indicated H_2_O_2_ treatments. Cells were subsequently incubated with 10 µM EdU‐containing complete medium for 2 h at 37°C. After EdU incorporation, cells were washed with PBS, fixed with 4% paraformaldehyde for 15 min, and permeabilized with 0.5% Triton X‐100 for 20 min. Incorporated EdU was detected using the supplied click‐reaction mixture according to the manufacturer's protocol, and nuclei were counterstained with Hoechst 33342. Fluorescence images were acquired under identical acquisition settings for all groups. Five randomly selected non‐overlapping fields were analyzed for each biological replicate using ImageJ. Cell proliferation was expressed as the percentage of EdU‐positive nuclei relative to the total number of Hoechst‐positive nuclei. Experiments were performed using three independent biological replicates, and measurements from multiple microscopic fields within the same biological replicate were averaged before statistical analysis.

### 4.10 Subcellular Fractionation

Mitochondrial and cytosolic fractions were prepared using a Cell Mitochondria Isolation Kit (Beyotime Biotechnology, Shanghai, China; Cat. No. C3601) according to the manufacturer's instructions. Briefly, ADSCs were harvested after the indicated treatments, washed with ice‐cold PBS, and resuspended in the supplied mitochondrial isolation buffer. Cells were homogenized on ice and subjected to differential centrifugation to separate the mitochondrial and cytosolic fractions. The mitochondrial pellet was collected and lysed for subsequent protein analysis, whereas the post‐mitochondrial supernatant was further clarified to obtain the cytosolic fraction. Whole‐cell lysates, mitochondrial fractions, and cytosolic fractions were analyzed by western blotting. TOMM20 was used as a mitochondrial marker and ACTB as a cytosolic marker to assess fraction purity. Experiments were performed in three independent biological replicates.

### 4.11 Genetically Encoded Mitochondrial Tracing

For genetically encoded mitochondrial tracing, donor ADSCs were transfected with a mitochondria‐targeted GFP reporter plasmid (mito‐GFP; Hunan Fenghui Biotechnology Co., Ltd., Changsha, China) using Lipofectamine 3000 Transfection Reagent (Invitrogen, Carlsbad, CA, USA; Cat. No. L3000015) according to the manufacturer's instructions. At 48 h after transfection, mitochondrial targeting of mito‐GFP was verified by immunofluorescence colocalization with TOMM20. Conditioned medium was then collected from mito‐GFP‐expressing ADSCs, and MVs and sEVs were isolated using the sequential centrifugation procedures described. The isolated vesicles were incubated with recipient NPCs, and GFP‐positive mitochondria‐associated cargo transferred to recipient cells was visualized by confocal fluorescence microscopy. Where indicated, vesicle membranes were additionally labelled with DiI to assess the spatial association between vesicular structures and mito‐GFP‐positive mitochondrial cargo. Experiments were performed in three independent biological replicates.

### 4.12 Isolation of MVs and sEVs

Conditioned medium was collected from ADSCs cultured in vesicle‐depleted medium containing 10% exosome‐depleted FBS (Gibco, A2720801). Cells and debris were removed by sequential centrifugation at 400×g for 10 min and 2000×g for 20 min. The supernatant was centrifuged at 10 000×g for 70 min at 4°C to collect the MV‐enriched fraction. MV pellets were washed with sterile PBS and centrifuged again at 10 000×g for 70 min. Final pellets were resuspended in PBS for downstream experiments. For sEV isolation, the supernatant after MV removal was filtered through a 0.22‐µm filter (Millipore, SLGP033RS) and ultracentrifuged at 100 000×g for 70 min at 4°C. Protein concentration was determined using a BCA protein assay kit (Beyotime, P0012). For experiments requiring matched MV input, MV loading was normalized either by total MV protein amount or by Annexin A1 signal, as indicated in the figure legends. For NTA‐normalized immunoblotting, equal numbers of NTA‐defined MV particles were loaded for each group. For per‐donor‐cell normalization, MVs were collected over the same 24‐h period from equal numbers of viable donor ADSCs, and equivalent donor‐cell‐normalized MV preparations were subjected to immunoblotting.

### 4.13 TEM and NTA

For conventional TEM characterization of extracellular vesicles, vesicle suspensions were fixed with 2% paraformaldehyde, loaded onto copper grids, negatively stained with 2% uranyl acetate, and examined using a TEM system (Hitachi, HT7700).

To examine the intravesicular ultrastructure of mitochondria‐containing MVs, ultrathin‐section TEM was performed by Wuhan Servicebio Technology Co., Ltd. (Wuhan, China) with minor modifications from previously described protocols. Briefly, freshly isolated MV pellets were fixed in 2.5% glutaraldehyde at 4°C, washed with phosphate‐buffered saline, and post‐fixed with 1% osmium tetroxide. Samples were dehydrated through a graded ethanol series, infiltrated with epoxy resin, and embedded for polymerization. Ultrathin sections were cut using an ultramicrotome, mounted onto copper grids, and stained with uranyl acetate and lead citrate. Sections were examined by TEM to visualize membrane‐bound intravesicular organelles and mitochondrial‐like ultrastructural features within MVs.

Particle size distribution and concentration were measured by NTA using a ZetaView PMX‐120 system (Particle Metrix). Samples were diluted in sterile phosphate‐buffered saline to an optimal particle concentration before acquisition, and data were analyzed using the manufacturer's software.

### 4.14 Vesicle Labeling, Uptake, and Mitochondrial Cargo Transfer Assays

For uptake assays, MVs were labeled with PKH26 (Sigma‐Aldrich, MINI26) according to the manufacturer's protocol and washed extensively with PBS to remove free dye. NPC lysosomes were labeled with LysoTracker Green (Invitrogen, L7526). For mitochondrial cargo transfer experiments, vesicle membranes were labeled with DiI (Invitrogen, D282), and mitochondrial cargo was labeled using Mito‐Tracker Green (Invitrogen, M7514). Labeled vesicles were incubated with NPCs for the indicated times, and vesicle uptake or mitochondrial cargo transfer was analyzed by confocal microscopy and flow cytometry.

### 4.15 Flow Cytometry Assays

For mitochondrial membrane potential analysis, cells or vesicle preparations were incubated with JC‐1 staining solution (Beyotime, C2006) according to the manufacturer's instructions. Mitochondrial membrane potential was evaluated by flow cytometry or fluorescence microscopy based on the ratio of JC‐1 aggregates to monomers. Intracellular reactive oxygen species were detected using 2',7'‐dichlorofluorescin diacetate (DCFH‐DA; Beyotime, S0033S). Flow cytometry was performed using a BD FACSCanto II flow cytometer, and data were analyzed using FlowJo software.

### 4.16 Mitochondrial Functional Assays

ATP content was measured using an enhanced ATP assay kit (Beyotime, S0026). MDA levels were measured using a lipid peroxidation MDA assay kit (Beyotime, S0131S). Protein carbonyl content was measured using a protein carbonyl colorimetric assay kit (Cayman Chemical, 10005020). All assays were performed according to the manufacturers' instructions, and values were normalized to protein content where appropriate.

### 4.17 Senescence‐Associated β‐Galactosidase Staining

ADSC senescence was assessed using a senescence‐associated β‐galactosidase staining kit (Beyotime, C0602). Cells were fixed and incubated with staining solution at 37°C without CO_2_. Positive cells were quantified from randomly selected fields.

### 4.18 IF Staining and Image Analysis

Cells or tissue sections were fixed in 4% paraformaldehyde (Servicebio, G1101), permeabilized with 0.1 to 0.3% Triton X‐100 (Sigma‐Aldrich, T8787), blocked with 5% bovine serum albumin (BSA; Sigma‐Aldrich, A7906), and incubated with primary antibodies overnight at 4°C. After washing, samples were incubated with Alexa Fluor 488‐ or Alexa Fluor 594‐conjugated secondary antibodies (Invitrogen, A11008 and A11012), and nuclei were stained with DAPI (Beyotime, C1002). Images were acquired using a laser‐scanning confocal microscope (Zeiss LSM 880 or Leica SP8). Colocalization coefficients and fluorescence intensity profiles were quantified using ImageJ.

### 4.19 Western Blotting

Cells, vesicles, or tissue samples were lysed in RIPA buffer (Beyotime, P0013B) supplemented with protease and phosphatase inhibitor cocktails (Roche, 04693159001 and 04906845001). Protein concentrations were determined by BCA assay. Equal amounts of protein were separated by SDS‐PAGE and transferred to PVDF membranes (Millipore, IPVH00010). Membranes were blocked with 5% nonfat milk and incubated with primary antibodies overnight at 4°C. After incubation with HRP‐conjugated secondary antibodies, signals were detected using enhanced chemiluminescence substrate (Biosharp, BL520A). Antibodies and catalog numbers are listed in Table S6, unless otherwise stated.

### 4.20 Co‐IP, SUMOylation, and Ubiquitination Assays

For Co‐IP, cells were lysed in IP buffer containing protease inhibitors. Lysates were incubated with the indicated antibodies or control IgG at 4°C overnight, followed by incubation with Protein A/G magnetic beads (Thermo Fisher Scientific, 88803). Beads were washed, and bound proteins were eluted and analyzed by western blotting. For SUMOylation assays, cells were lysed in buffer supplemented with 20 mM N‐ethylmaleimide (NEM; Sigma–Aldrich, E3876) to inhibit deSUMOylation. GFP‐HSPA9 or endogenous HSPA9 was immunoprecipitated and immunoblotted with anti‐HA, anti‐SUMO1, or anti‐SUMO2/3 antibodies. For ubiquitination assays, cells were treated with MG132 (Selleck Chemicals, S2619) when indicated, HSPA9 was immunoprecipitated, and ubiquitinated HSPA9 was detected using an anti‐ubiquitin antibody. Antibodies used in these assays are listed in Table S6 unless otherwise stated.

### 4.21 Protein stability assays

For CHX chase assays, ADSCs expressing GFP‐HSPA9 wild‐type or mutant proteins were treated with CHX for the indicated times. Cell lysates were collected and analyzed by western blotting. For proteasome inhibition experiments, cells were treated with MG132 or dimethyl sulfoxide (DMSO; Sigma–Aldrich, D2650) before protein extraction.

### 4.22 TMT‐Based Quantitative Proteomics and Bioinformatics

MV samples were subjected to tandem mass tag (TMT)‐based quantitative proteomic analysis. Protein concentration and quality were assessed before digestion. Proteins were digested with sequencing‐grade trypsin (Promega, V5280), desalted using C18 tips (Millipore, ZTC18M096), and labeled with TMT10plex reagents (Thermo Fisher Scientific, 90111) according to the manufacturers’ instructions. The labeled peptides were pooled and fractionated using an XBridge Peptide BEH C18 column (Waters, 186003581). Liquid chromatography‐tandem mass spectrometry (LC‐MS/MS) was performed on an Orbitrap mass spectrometer (Thermo Fisher Scientific). Raw files were searched against the UniProt human proteome database. Peptide and protein identifications were controlled at an FDR of <1%. Three independent biological replicates were analyzed per group. Protein abundance values were median‐normalized across samples. Differential abundance was assessed using Student's *t*‐test, and *p*‐values were adjusted for multiple testing using the Benjamini–Hochberg procedure. Proteins with a fold change ≥1.20 or ≤0.833 and an adjusted FDR <0.05 were considered differentially abundant. Data quality and sample reproducibility were assessed by unsupervised hierarchical clustering, normalized protein abundance distributions, Pearson correlation analysis, and principal component analysis. Gene Ontology and Reactome enrichment analyses were performed using the clusterProfiler package in R, and gene set enrichment analysis was performed using ranked protein lists. The mass spectrometry proteomics data generated in this study have been deposited in the ProteomeXchange Consortium via the iProX partner repository with the dataset identifier PXD072918.

### 4.23 Recombinant Protein Expression and Purification

Recombinant human HSPA9 and PINK1 proteins were expressed and purified by a commercial service. HSPA9 (amino acids 1 to 679; UniProt P38646) and PINK1 (amino acids 111 to 581; UniProt Q9BXM7) were expressed in E. coli T7E cells with N‐His‐SUMO and C‐His tags. Protein expression was induced with 1 mM IPTG (Amresco, 0487) at 18°C for 12 h. Cells were collected, sonicated in PBS, and centrifuged to remove debris. His‐tagged proteins were purified using Ni‐NTA agarose (Invitrogen, R90101), dialyzed into PBS, and assessed by Coomassie brilliant blue staining before surface plasmon resonance assays.

### 4.24 SPR

SPR assays were performed by Wuhan Mabnus Biotech Co., Ltd. (Wuhan, China) using a Biacore T200 system (Cytiva) with a CM5 sensor chip (Cytiva, BR‐1005‐30) at 25°C. Recombinant human HSPA9 and PINK1 proteins used for SPR analysis were expressed and purified by Wuhan Mabnus Biotech Co., Ltd. (Wuhan, China). HSPA9 was immobilized on channel 3 of the CM5 sensor chip by amine coupling using an Amine Coupling Kit (Cytiva, BR‐1000‐50), with channel 1 used as the reference channel. HSPA9 was diluted to 20 µg/mL in 10 mM sodium acetate buffer (pH 4.5; Cytiva, BR‐1003‐50) and immobilized to approximately 3000 response units. PINK1 was injected as the analyte at 0, 62.5, 125, 250, 500, and 1000 nM in HBS‐EP running buffer. The association and dissociation times were 180 and 120 s, respectively. The chip surface was regenerated with 10 mM glycine (pH 1.5). Sensorgrams were reference‐subtracted and fitted using a 1:1 binding model and a steady‐state affinity model in Biacore T200 Evaluation Software.

### 4.25 Split‐Ubiquitin Yeast Two‐Hybrid Assay

The split‐ubiquitin membrane yeast two‐hybrid assay was performed by Nanjing Consure Biotechnology Co., Ltd. (Nanjing, China) to validate the interaction between HSPA9 and PINK1. Briefly, PINK1 was cloned into the pBT3‐STE bait vector, and HSPA9 was cloned into the pPR3‐N prey vector. The pBT3‐STE‐PINK1 + pPR3‐N‐HSPA9 combination was used as the experimental group. The pBT3‐STE‐PINK1 + pPR3‐N combination was used to assess bait autoactivation. The pTSU2‐APP + pNubG‐Fe65 pair and the pTSU2‐APP + pPR3‐N pair were used as positive and negative controls, respectively. The indicated bait and prey plasmids were co‐transformed into NMY51 competent yeast cells using the lithium acetate/polyethylene glycol method. Transformed yeast cells were first selected on SD/‐Trp/‐Leu plates at 30°C for 48–96 h to confirm successful co‐transformation. Single colonies were then cultured in SD/‐Trp/‐Leu medium, adjusted to an initial optical density at 600 nm of 0.2, serially diluted from 10^0^ to 10^−^^3^, and spotted onto SD/‐Trp/‐Leu and SD/‐Trp/‐Leu/‐His/‐Ade plates. For stringent interaction screening, SD/‐Trp/‐Leu/‐His/‐Ade plates supplemented with 3‐amino‐1,2,4‐triazole (3‐AT; Coolaber, CA1311) were also used where indicated. Yeast growth on SD/‐Trp/‐Leu plates indicated successful plasmid transformation, whereas growth on SD/‐Trp/‐Leu/‐His/‐Ade plates indicated protein‐protein interaction‐dependent reporter activation. The absence of growth in the bait autoactivation and negative control groups confirmed that PINK1 did not exhibit detectable autoactivation under these conditions. SD/‐Leu/‐Trp medium (Coolaber, PM2221), SD/‐Leu/‐Trp/‐His/‐Ade medium (Coolaber, PM2111), YPDA medium (Coolaber, YT0007), 3‐amino‐1,2,4‐triazole (3‐AT; Coolaber, CA1311), NMY51 competent yeast cells (Shanghai Weidi Biotechnology, YC1040S) and agar powder (Servicebio, G3054) were used for yeast transformation, selection and interaction screening.

### 4.26 AlphaFold3 Modeling and Molecular Dynamics Simulation

HSPA9 and PINK1 sequences were submitted to AlphaFold3 for complex structure prediction. The best‐ranked HSPA9‐PINK1 complex was selected for all‐atom molecular dynamics simulation. Simulations were performed using GROMACS 2022.5 with the AMBER14SB force field and TIP3P water model. The complex was placed in a solvent box with at least 1.2 nm padding and neutralized with Na+/Cl‐ ions at 0.15 M. Energy minimization was performed using the steepest descent algorithm, followed by NVT and NPT equilibration at 300 K and 1 bar. A 100‐ns production simulation was performed with a 2‐fs time step. Long‐range electrostatic interactions were calculated using Particle Mesh Ewald, and bond constraints were applied using the LINCS algorithm. Root mean square deviation, root mean square fluctuation, radius of gyration, principal component analysis, free energy landscape, secondary structure, and Ramachandran analyses were performed using GROMACS and UCSF ChimeraX. Binding free energy was calculated from the last 10 ns of the trajectory using MM‐GBSA with gmx_MMPBSA, and per‐residue energy decomposition was performed to identify interface hotspot residues.

### 4.27 Rat Caudal Disc Puncture Model and Intradiscal Injection

All animal procedures were approved by the Institutional Animal Care and Use Committee of Huazhong University of Science and Technology (No. 4571) and were performed in accordance with institutional guidelines for animal care and use. SD rats were purchased from Shulaibao (Wuhan) Biotechnology Co., Ltd. (Wuhan, China) and aged 6–8 weeks were used. Male rats were selected to maintain sex consistency with our established rat caudal disc puncture model. SD rats were housed under standard conditions with free access to food and water. For all in vivo experiments, each experimental group included five rats (n = 5), with one treated Co8/9 disc per animal considered as one independent biological replicate. The sample size was selected based on prior experience with the established rat caudal IVDD model [6, 39, 40], and no formal a priori power calculation was performed. Animals were allocated to experimental groups using a random‐number‐based procedure before treatment. Group allocation was performed independently of subsequent imaging and histological assessment. Because the investigator performing intradiscal administration necessarily had access to the treatment preparations, blinding was not feasible during treatment administration. However, MRI grading, radiographic and micro‐CT measurements, histological scoring, and quantitative tissue‐image analyses were performed with group identities masked until completion of data acquisition and quantification. Each rat, with one treated Co8/9 disc, was considered an independent biological replicate. A rat caudal IVDD model was established by needle puncture as previously described with minor modifications [6, 7]. Briefly, rats were anesthetized by intraperitoneal injection of pentobarbital sodium, and the caudal intervertebral discs were identified by palpation and radiographic guidance. The Co8/9 caudal intervertebral disc was selected as the target segment for puncture and subsequent intradiscal treatment. After disinfection with povidone‐iodine and 75% ethanol, a sterile needle was inserted percutaneously into the Co8/9 disc through the annulus fibrosus, parallel to the endplate, until the nucleus pulposus region was reached. The needle was gently rotated and retained briefly to induce reproducible disc injury before withdrawal. Two weeks after puncture, rats received intradiscal injection of 20 µL PBS or MV (50 µg/mL) preparations into the same Co8/9 disc using a microsyringe. For therapeutic comparison experiments, MVnormal, MV_stress,_ or MV_modified_ were injected according to the experimental design. The injection volume and MV dose were kept identical among groups. After injection, the needle was retained briefly before withdrawal to minimize leakage. Rats were then allowed to recover on a heating pad and were monitored until full recovery from anesthesia. Disc degeneration was evaluated at the indicated time points after puncture and MV treatment. T2‐weighted MRI was performed to assess disc hydration and signal intensity, and the degree of degeneration was evaluated using the Pfirrmann grading system. X‐ray imaging was performed to assess disc‐space narrowing. Micro‐CT was used to reconstruct the caudal motion segment and quantify the DHI. Disc height was measured at the midline and at points 25% of the disc width from the midline on both sides. The mean disc height was normalized to the mean height of the adjacent vertebral bodies to calculate DHI. Changes in DHI were expressed relative to the corresponding pre‐puncture or internal control values where applicable. At the experimental endpoint, rats were euthanized, and caudal disc tissues containing the treated Co8/9 segment were harvested for histological and immunostaining analyses. Specimens were fixed in 4% paraformaldehyde, decalcified, embedded in paraffin, and sectioned along the midsagittal plane. H&E staining, Masson staining, and SOFG staining were performed to evaluate nucleus pulposus structure, annulus fibrosus organization, and extracellular matrix integrity. Histological degeneration was scored according to a previously established rat disc degeneration grading system [40].

### 4.28 Assessment of Xenogeneic Immune Responses to Human ADSC‐Derived MVs

To assess the potential xenogeneic immune response induced by human ADSC‐derived MVs, male SD rats were purchased from Hubei Biont Biological Technology Co., Ltd. Animals were acclimatized for 7 days under specific pathogen‐free conditions before experimentation and were randomly allocated to three groups (n = 5 rats per group): PBS, human ADSC‐derived MVnormal (MV_human_), and species‐matched rat ADSC‐derived MV (MV_rat_) groups. Rat ADSCs were isolated from rat adipose tissue using the same procedure described above for human ADSCs and cultured under identical conditions. Rat ADSC morphology was examined by light microscopy, and the mesenchymal phenotype was further characterized by flow cytometry using anti‐rat CD73‐PE (BioLegend, San Diego, CA, USA; Cat. No. 551124) and anti‐rat CD45‐FITC antibodies (Invitrogen, Carlsbad, CA, USA; Cat. No. 11‐0461‐82). Rat ADSC‐derived MVs were collected from conditioned medium using the same sequential centrifugation protocol as that used for human ADSC‐derived MVs and characterized by transmission electron microscopy and nanoparticle tracking analysis before administration.

Rats received a single intradiscal injection of 20 µL PBS, human MV_normal_, or rat ADSC‐derived MVs at an MV concentration of 50 µg/mL. Seven days after MV administration, peripheral blood, injected caudal disc tissues, and major organs were collected for assessment of local and systemic immune responses. Blood samples were allowed to clot at room temperature for 30 min and centrifuged at 3000 × g for 10 min at 4°C. Serum was collected and stored at −80°C until analysis. Serum IL‐6 and TNF‐α concentrations were measured using a Rat IL‐6 ELISA Kit (Boster Biological Technology Co., Ltd., Wuhan, China; Cat. No. EK0412) and a Rat TNF‐α ELISA Kit (Boster Biological Technology Co., Ltd.; Cat. No. EK0526), respectively, according to the manufacturer's instructions. Absorbance was measured at 450 nm using a microplate reader, and cytokine concentrations were calculated from the corresponding standard curves.

Local leukocyte infiltration was evaluated by IHC staining of disc sections using an anti‐CD45 antibody (Proteintech, Wuhan, China; Cat. No. 20103‐1‐AP). Paraffin sections were subjected to antigen retrieval, blocked, incubated with the primary antibody overnight at 4°C, and subsequently developed using an HRP/DAB detection system. CD45‐positive staining was quantified from randomly selected non‐overlapping fields using ImageJ, with the mean value from multiple fields used as one biological replicate. The heart, liver, spleen, lung, and kidney were harvested at the same endpoint, fixed in 4% paraformaldehyde for 24 h, paraffin‐embedded, sectioned, and subjected to hematoxylin and eosin staining (Servicebio, Wuhan, China; Cat. No. G1005) to evaluate potential systemic histopathological alterations.

### 4.29 MRI, X‐Ray, Micro‐CT, and In Vivo Fluorescence Imaging

MRI was performed to assess disc hydration and degeneration using T2‐weighted sequences. Pfirrmann grades were assigned by blinded observers. X‐ray imaging was used to evaluate disc height, and the DHI was calculated from radiographs. Micro‐CT was performed to reconstruct rat caudal discs and assess structural changes. For in vivo tracking, MVs were labeled with DiR dye (Yeasen, 40757ES25) according to the manufacturer's instructions and detected using an IVIS Spectrum imaging system (PerkinElmer).

### 4.30 Histology, IHC, and Tissue IF

Rat caudal discs were fixed in 4% paraformaldehyde, decalcified in 10% EDTA (Servicebio, G1105), embedded in paraffin, and sectioned. Sections were stained with hematoxylin and eosin (Servicebio, G1005), Masson trichrome (Servicebio, G1006), and SOFG (Servicebio, G1053). Histological scores were evaluated by blinded observers. For IHC, sections were subjected to antigen retrieval, blocked, and incubated with primary antibodies against collagen I (Proteintech, 14695‐1‐AP), aggrecan (Proteintech, 13880‐1‐AP), or NLRP3 (Proteintech, 19771‐1‐AP), followed by HRP‐conjugated secondary antibody and DAB substrate (Servicebio, G1212). For tissue IF, sections were incubated with rabbit anti‐cGAS antibody (Proteintech, 26416‐1‐AP) and mouse anti‐STING antibody (Proteintech, 66680‐1‐Ig), followed by species‐specific fluorescent secondary antibodies and DAPI counterstaining.

### 4.31 Experimental Replicates and Definition of Sample Size

Unless otherwise stated, quantitative in vitro experiments were performed using three biological replicates from three independent experiments. A biological replicate was defined as an independently prepared and treated cell culture or MV preparation generated in a separate experiment. Multiple wells, repeated measurements, microscopic fields, or repeated instrument acquisitions derived from the same biological sample were considered technical replicates and were averaged to generate a single value for the corresponding biological replicate; technical replicates were not treated as independent n values. For key experiments using primary human ADSCs or NPCs, three independent donors were included as biological replicates (n = 3), unless otherwise indicated. For animal experiments, one rat with one treated Co8/9 disc was considered one biological replicate. The exact number of biological replicates and independent experiments is specified in the corresponding figure legends.

### 4.32 Statistical Analysis

Data are presented as mean ± standard deviation (s.d.). Statistical analyses were performed using GraphPad Prism 9. For comparisons between two independent groups, an unpaired two‐tailed Student's *t*‐test was used. For comparisons among three or more groups with one experimental factor, one‐way analysis of variance (ANOVA) followed by Tukey's multiple‐comparisons test was used. Dunnett's multiple‐comparisons test was applied when multiple treatment groups were compared with a single control group. For experiments involving two independent factors, such as treatment and time, two‐way ANOVA followed by Sidak's or Tukey's multiple‐comparisons test was used when appropriate. The specific statistical test used for each experiment is indicated in the corresponding figure legends. P values less than 0.05 were considered statistically significant.

## Author Contributions

Z. O., M.Z., and Z.L. conceived and designed the study. Z.O., M.Z., Z.L., L.P., B.T., H.L., H.W., D.W., Y.D., Z.D., S.Z., Y.J., Y.Q., G.X., L.H., G.L., S.Z., L.T., and K.W. performed the experiments and analyzed the data. X.F., C.Y., and Z.L. supervised the project. Z.O., M.Z., Z.L., and L.P. drafted the manuscript. All authors participated in the interpretation of the data and production of the final manuscript.

## Conflicts of Interest

The authors declare no conflicts of interest.

## Supporting information




**Supporting File**: advs78392‐sup‐0001‐SuppMat.docx.

## Data Availability

The data that support the findings of this study are openly available in ProteomeXchange at http://proteomecentral.proteomexchange.org/cgi/GetDataset?ID=PXD072918, reference number PXD072918.
