# Supplementary material for: Donor‐Cell Senescence Redirects PINK1‐Marked Mitochondrial Cargo From Mitophagy to Microvesicle Transfer Through SUMO3‐Stabilized HSPA9
Source: Adv Sci (Weinh). 2026 Oct 25:e78392. Online ahead of print. doi: 10.1002/advs.78392 (PMC13696783; doi:10.1002/advs.78392)
Supplement: Supplementary file 1 [file ADVS-9999-e78392-s001.docx]

Supplementary Information

**Donor-cell senescence redirects PINK1-marked mitochondrial cargo from mitophagy to microvesicle transfer through SUMO3-stabilized HSPA9**

Zixuan Ou^1,#^, Mingke Zhao^1,#^, Zhiwei Ling^1,#^, Lianqi Peng^2,#^, Bide Tong^1^, Huaizhen Liang^1^, Hongchuan Wang^1^, Di Wu^1^, Yifan Du^1^, Zhi Du^1^, Shihao Zhang^1^, Yuyuan Jia^1^, Yifan Qian^1^, Gan Xu^1^, Liuliang He^1^, Gang Li^1^, Shun Zhang^1^, Lei Tan^1^, Kun Wang^1^, Xiaobo Feng^1,*^, Cao Yang^1,*^, Zhiwei Liao^1,*^

^#^These authors contributed equally: Zixuan Ou, Mingke Zhao, Zhiwei ling, Lianqi Peng.

^*^Correspondence to: Xiaobo Feng [(](mailto:(fengxiaobo1204@163.com))[fengxiaobo@hust.edu.cn)](mailto:(fengxiaobo1204@163.com)), Cao Yang [(caoyangunion@hust.edu.cn)](mailto:(caoyangunion@hust.edu.cn)) or Zhiwei Liao (lzwhust@hust.edu.cn).

**Table. S1. Clinical characteristics of human nucleus pulposus tissue donors (n = 6).**

| Case Number | Gender | Age (Years) | Diagnosis | Segment | MRI Grade | Operation Method |
| --- | --- | --- | --- | --- | --- | --- |
| Case 1 | Male | 18 | Idiopathic scoliosis | L1-L2 | II | Open spine surgery |
| Case 2 | Female | 14 | Idiopathic scoliosis | L1-L2 | I | Open spine surgery |
| Case 3 | Male | 18 | Idiopathic scoliosis | T12-L1 | I | Open spine surgery |
| Case 4 | Female | 23 | Idiopathic scoliosis | L1-L2 | II | Open spine surgery |
| Case 5 | Male | 15 | Idiopathic scoliosis | L1-L2 | I | Open spine surgery |
| Case 6 | Female | 10 | Idiopathic scoliosis | T12-L1 | I | Open spine surgery |

**Table. S2. Clinical characteristics of human adipose tissue donors (n = 6).**

| Case Number | Gender | Age (Years) | Weight | Indication for liposuction | Liposuction site |
| --- | --- | --- | --- | --- | --- |
| Case 1 | Female | 37 | 60 kg | Elective cosmetic liposuction | Thigh |
| Case 2 | Female | 44 | 58 kg | Elective cosmetic liposuction | Thigh |
| Case 3 | Female | 42 | 55 kg | Elective cosmetic liposuction | Thigh |
| Case 4 | Male | 28 | 75 kg | Elective cosmetic liposuction | Thigh |
| Case 5 | Male | 28 | 80 kg | Elective cosmetic liposuction | Thigh |
| Case 6 | Male | 29 | 82 kg | Elective cosmetic liposuction | Thigh |

**Table. S3. siRNA sequences used in this study**

| Gene Name | Sense sequence | Antisense sequence |
| --- | --- | --- |
| si-PINK1 | GCCGCAAAUGUGCUUCAUCUA | UAGAUGAAGCACAUUUGCGGC |
| si-HSPA9 | AGACAAAUCAGAAGACAAATT | UUUGUCUUCUGAUUUGUCUTT |
| si-SUMO3 | CGUGGUGCAGUUCAAGAUCAA | UUGAUCUUGAACUGCACCACG |
| si-cGAS | CUAGCAACUUAAUUGACAATT | UUGUCAAUUAAGUUGCUAGTT |
| si-STING | CUGGCAUGGUCAUAUUACATT | UGUAAUAUGACCAUGCCAGTT |
| si-scramble | UUCUCCGAACGUGUCACGUTT | ACGUGACACGUUCGGAGAATT |

**Table. S4. Primers for qRT-PCR in this study**

| Gene Name | Forward Primer (5′-3′) | Reverse Primer (5′-3′) |
| --- | --- | --- |
| IL6 | AGACAGCCACTCACCTCTTCAG | TTCTGCCAGTGCCTCTTTGCTG |
| CXCL8 | GTCCTTGTTCCACTGTGCCT | GCTTCCACATGTCCTCACAA |
| CCL2 | AGAATCACCAGCAGCAAGTGTCC | TCCTGAACCCACTTCTGCTTGG |
| ACTB | CCTGTACGCCAACACAGTGC | ATACTCCTGCTTGCTGATCC |
| GAPDH | CTCCTCCACCTTTGACGCTG | TCCTCTTGTGCTCTTGCTGG |

**Table. S5. Primers for qPCR in this study**

| Gene Name | Forward Primer (5′-3′) | Reverse Primer (5′-3′) |
| --- | --- | --- |
| β-globin | TGGATGCTCTCATCAGGACAG | AAAGGTGCCCTTGAGGTTGTC |
| ND1 | CCCTAAACCCGCCACATCT | GAGCGATGGTGAGAGCTAAGGT |
| ND2 | CCCACACACACACCGCTAAC | ATTGCTTTCGTGGTTATCTGCT |
| COX1 | TCGCCGACCGTTGACTATTCT | ATGAGCCGCAGGTACTGTATC |
| ATP6 | ACCAACACCTCTTTACAGTGAA | TGGTTGTCCTGCGCTATCTG |

**Table. S6. Information of antibodies**

| Antibodies | Source | Identifier |
| --- | --- | --- |
| anti-HSPA9 | Proteintech | 14887-1-AP |
| anti-γH2A.X (S139) | Proteintech | 83307-2-RR |
| anti-p-p53 | Proteintech | 28961-1-AP |
| anti-Annexin A1 | Proteintech | 21990-1-AP |
| anti-HSP60 | Proteintech | 15282-1-AP |
| anti-VDAC1 | Proteintech | 10866-1-AP |
| anti-TSG101 | Proteintech | 28283-1-AP |
| anti-Calnexin | Proteintech | 10427-2-AP |
| anti-CD9 | Proteintech | 20597-1-AP |
| anti-MLC1 | Proteintech | 26038-1-AP |
| anti-ROCK1 | Proteintech | 21850-1-AP |
| anti-RhoA | Proteintech | 10749-1-AP |
| anti-ARF6 | Proteintech | 20225-1-AP |
| anti-SUMO1 | Proteintech | 10329-1-AP |
| anti-SUMO2/3 | Zenbio | 660001 |
| Anti-ubiquitin | Proteintech | 10201-2-AP |
| anti-GFP | Proteintech | 50430-2-AP |
| anti-HA | Proteintech | 51064-2-AP |
| anti-FLAG | Proteintech | 20543-1-AP |
| anti-FLAG | Proteintech | 66008-4-Ig |
| anti-TOMM20 | Proteintech | 11802-1-AP |
| anti-TOMM20 | Proteintech | 66777-1-Ig |
| anti-LC3 | Proteintech | 14600-1-AP |
| anti-PINK1 | Affinity | DF7742 |
| anti-Parkin | Affinity | AF0235 |
| anti-Parkin | Abnova | MAB12322 |
| anti-SQSTM1 | Proteintech | 80294-1-RR |
| anti-ACTB | Proteintech | 66009-1-Ig |
| anti-NLRP3 | Proteintech | 19771-1-AP |
| anti-aggrecan | Proteintech | 13880-1-AP |
| anti-collagen I | Proteintech | 14695-1-AP |
| anti-cGAS | Proteintech | 26416-1-AP |
| anti-STING | Proteintech | 66680-1-Ig |
| anti-p21 | Proteintech | 10355-1-AP |
| anti-CD45 | Proteintech | 20103-1-AP |
| anti-dsDNA | Arigo | ARG40774 |
| CD73-PE | BioLegend | 344004 |
| CD73-PE | BioLegend | 551124 |
| CD90-FITC | BioLegend | 328108 |
| CD105-FITC | BioLegend | 323204 |
| CD45-FITC | Invitrogen | 11-0461-82 |
| HLA-DR-PE | BioLegend | 307606 |
| anti-rabbit IgG, HRP-linked antibody | Cell Signaling Technology | 7074 |
| anti-mouse IgG, HRP-linked antibody | Cell Signaling Technology | 7076 |
| Rabbit IgG control polyclonal antibody | Proteintech | 30000-0-AP |
| 594-Goat Anti-Rabbit Recombinant Secondary Antibody | Proteintech | RGAR004 |
| 488-Goat Anti-Mouse Recombinant Secondary Antibody | Proteintech | RGAM002 |
| Mouse IgG | Proteintech | B900620 |

**Supplementary Figure 1. Isolation and phenotypic characterization of human ADSCs.** (a) Schematic illustration of the isolation of human ADSCs from adipose tissues; (b–e) Representative flow cytometry profiles and quantitative analyses of ADSC surface markers, including CD73, CD90, CD105 and HLA-DR; (f) Representative images showing the morphology of cultured ADSCs and their osteogenic and adipogenic differentiation capacities, as assessed by Alizarin Red S and Oil Red O staining, respectively. Scale bar, 100 μm. Quantitative data are presented as mean ± s.d. from three biological replicates obtained in three independent experiments (n = 3). Statistical significance was determined by unpaired two-tailed Student’s t-test.

**Supplementary Figure 2. Additional validation of ADSC senescence and the cGAS-STING-dependent effects of senescent ADSC-derived MVs on recipient NPCs.** (a) Quantification of mitochondrial membrane potential by JC-1 flow cytometry in H_2_O_2_-injured NPCs treated with increasing concentrations of MV_normal_; (b) Quantification of cGAS, STING and NLRP3 protein expression in H_2_O_2_-injured NPCs treated with increasing concentrations of MV_normal_; (c) Quantification of γH2A.X(S139), p-p53 and p21 protein expression in ADSCs exposed to the indicated concentrations of H_2_O_2_; (d) Quantification of γH2A.X(S139) immunofluorescence intensity in ADSCs after H_2_O_2_ treatment; (e) qRT-PCR analysis of the representative SASP factors IL6, CXCL8 and CCL2 in control and H_2_O_2_-treated ADSCs; (f) Representative EDU fluorescence images and quantification of EDU-positive ADSCs after H_2_O_2_ treatment. Scale bar, 100 μm; (g) Representative confocal images showing the uptake and lysosome-associated localization of PKH26-labelled MV_normal_ and MV_stress_ in recipient NPCs. Lysosomes were labelled with LysoTracker. Scale bar, 20 μm; (h) Quantification of cGAS, STING and NLRP3 protein expression in H_2_O_2_-injured NPCs treated with MV_normal_ or MV_stress_; (i) WB analysis of cGAS, STING and NLRP3 in MVstress-treated NPCs transfected with si-scramble, si-cGAS or si-STING; (j) Quantification of cGAS, STING and NLRP3 protein expression shown in i; (k) Representative DCFH flow cytometry histograms showing ROS levels in MV_stress_-treated NPCs after cGAS or STING silencing; (l) Quantification of DCFH-positive cells shown in k; (m) Cellular mtDNA abundance in control and H_2_O_2_-treated donor ADSCs, quantified using ND1, ND2, COX1 and ATP6 and normalized to the nuclear β-globin gene; (n) Relative abundance of MV-associated mtDNA, including ND1, ND2, COX1 and ATP6, in MVs derived from control or H₂O₂-treated ADSCs under equal NTA-defined MV input; (o) Quantification of mitochondrial membrane potential by JC-1 flow cytometry in H_2_O_2_-injured NPCs treated with MVnormal or MVstress; (p) Quantification of ROS-positive cells by DCFH flow cytometry in the indicated groups; (q) Quantification of TOMM20-DNA colocalization in recipient NPCs by Pearson’s correlation coefficient. Quantitative data are presented as mean ± s.d. from three biological replicates obtained in three independent experiments (n = 3). Two-group comparisons were analyzed using an unpaired two-tailed Student’s t-test, whereas comparisons among three or more groups were analyzed by one-way ANOVA followed by multiple-comparisons testing.

**Supplementary Figure 3. Characterization of rat ADSCs and ADSC-derived MVs, assessment of xenogeneic immune responses, and additional in vivo analyses of IVDD.** (a) Schematic illustration of rat ADSC isolation and representative morphology of cultured rat ADSCs. Scale bar, 100 μm; (b,c) Representative flow cytometry profile and quantitative analysis of CD73 expression in rat ADSCs; (d,e) Representative flow cytometry profile and quantitative analysis of CD45 expression in rat ADSCs; (f) NTA showing the size distribution of rat ADSC-derived MVs; (g) Representative TEM image of rat ADSC-derived MVs. Scale bar, 500 nm; (h) Representative IHC images and quantification of CD45-positive leukocyte infiltration in rat caudal discs after intradiscal administration of PBS, rat ADSC-derived MVs (MV_rat_) or human ADSC-derived MVs (MV_human_). Scale bar, 100 μm; (i) Representative H&E staining of the liver, spleen, kidney, heart and lung after the indicated treatments. Scale bar, 100 μm; (j,k) Serum IL-6 and TNF-α concentrations measured by ELISA after intradiscal administration of PBS, MV_rat_ or MV_human_; (l) Histological scores of rat caudal discs in the Control, IVDD, MV_normal_ and MV_stress_ groups; (m) Representative line-scan fluorescence intensity profiles of cGAS and STING in rat disc tissues from the indicated groups. For rat ADSC characterization, quantitative data were obtained from three independent biological replicates (n = 3). For in vivo analyses, n = 5 rats per group, with one treated disc per rat considered one independent biological replicate. Data are presented as mean ± s.d. Statistical significance was determined by one-way ANOVA followed by multiple-comparisons testing.

**Supplementary Figure 4. Genetically encoded tracing confirms mitochondrial cargo transfer through ADSC-derived MVs.** (a) Representative confocal images showing mitochondrial targeting of the genetically encoded mito-GFP reporter in donor ADSCs. Mitochondria were immunostained for TOMM20, and colocalization of GFP with TOMM20 confirmed mitochondrial localization of the reporter. Scale bar, 50 μm; (b) Representative confocal images of recipient NPCs after incubation with sEVs or MVs isolated from mito-GFP-expressing donor ADSCs. Vesicle membranes were labelled with DiI, and GFP-positive signals represent genetically labelled mitochondria-associated cargo transferred from donor ADSCs. Scale bar, 20 μm. Right, quantification of GFP fluorescence intensity in recipient NPCs. Quantitative data are presented as mean ± s.d. from three biological replicates obtained in three independent experiments (n = 3). Statistical significance was determined by unpaired two-tailed Student’s t-test.

**Supplementary Figure 5. Proteomic profiling and biochemical validation of mitochondrial cargo remodeling in senescent ADSC-derived MVs.** (a) Coomassie brilliant blue staining of ADSC cell lysates, sEVs and MVs under equal total protein loading (20 μg per sample); (b) Unsupervised hierarchical clustering of median-normalized TMT proteomic profiles from MV_normal_ and MV_stress_. Three independent biological replicates were analyzed per group; (c) Distribution of normalized protein abundance across the six proteomic samples; (d) Pearson correlation matrix showing reproducibility among biological replicates; (e) Principal component analysis of MV_normal_ and MV_stress_ proteomic profiles; (f) GO biological process enrichment analysis of differentially abundant proteins. Differential abundance was defined as fold change ≥1.20 or ≤0.833 together with a Benjamini-Hochberg-adjusted FDR <0.05; (g) Representative NTA size-distribution profiles of MV_normal_ and MV_stress_ collected from equal numbers of viable donor ADSCs over the same collection period; (h) Quantification of MV particle output from normal and H_2_O_2_-treated donor ADSCs; (i) Quantification of MLC1, ROCK1, RhoA and ARF6 expression in donor ADSCs, corresponding to Fig. 3o; (j) Quantification of VDAC1, PINK1, TOMM20, TSG101, Calnexin, Annexin A1 and CD9 expression in donor ADSCs, corresponding to Fig. 3p; (k) Coomassie brilliant blue staining confirming equal total MV protein loading (20 μg per sample) between MV_normal_ and MV_stress_; (l) Quantification of VDAC1, PINK1, TOMM20, TSG101, Annexin A1 and CD9 in MV_normal_ and MV_stress_ under equal total MV protein loading, corresponding to Fig. 3q; (m) Quantification of PINK1, TOMM20, VDAC1 and Annexin A1 in MV_normal_ and MV_stress_ under comparable Annexin A1 loading, corresponding to Fig. 3r; (n) WB analysis of Annexin A1, VDAC1, PINK1 and TOMM20 in MV_normal_ and MV_stress_ under equal NTA-defined particle input; (o) Quantification of the proteins shown in n; (p) WB analysis of Annexin A1, VDAC1, PINK1 and TOMM20 using MV samples normalized to equal numbers of viable donor ADSCs over the same collection period; (q,r) Quantification of the proteins shown in p. Unless otherwise indicated, quantitative data are presented as mean ± s.d. from three biological replicates obtained in three independent experiments (n = 3). Proteomic analyses used n = 3 independent biological replicates per group. Statistical significance for two-group comparisons was determined by unpaired two-tailed Student’s t-test.

**Supplementary Figure 6. Subcellular distribution of HSPA9 and orthogonal validation of HSPA9-dependent mitochondrial cargo loading into MVs.** (a) Quantification of HSPA9 expression in ADSCs treated with or without H_2_O_2_, corresponding to Fig. 4c; (b) Quantification of HSPA9 and Annexin A1 levels in MV_normal_ and MV_stress_, corresponding to Fig. 4d; (c-e) WB analysis of HSPA9, TOMM20, PINK1 and ACTB in whole-cell lysates (WCL), cytosolic fractions (Cyto) and mitochondrial fractions (Mito) from control and H_2_O_2_-treated ADSCs. TOMM20 and ACTB were used as mitochondrial and cytosolic fraction markers, respectively; (f,g) WB analysis and quantification of HSPA9 knockdown efficiency in ADSCs under basal or H_2_O_2_-treated conditions; (h-j) Quantification of VDAC1, PINK1 and Annexin A1 in ADSC-derived MVs after HSPA9 knockdown, corresponding to Fig. 4l. MV input was adjusted to comparable Annexin A1 levels; (k) WB analysis of VDAC1, PINK1 and Annexin A1 in ADSC-derived MVs under equal NTA-defined particle input after HSPA9 knockdown; (l-n) Quantification of the proteins shown in k; (o) WB analysis of VDAC1, PINK1 and Annexin A1 in MVs normalized to equal numbers of viable donor ADSCs over the same collection period; (p-r) Quantification of the proteins shown in o. Quantitative data are presented as mean ± s.d. from three biological replicates obtained in three independent experiments (n = 3). Two-group comparisons were analyzed using an unpaired two-tailed Student’s t-test, and comparisons among three or more groups were analyzed by one-way ANOVA followed by multiple-comparisons testing.

**Supplementary Figure 7. Global modulation of cellular SUMOylation is associated with altered HSPA9-cargo interactions and mitochondrial cargo loading into MVs.** (a) Reactome pathway enrichment analysis of differentially abundant proteins, highlighting SUMOylation-related pathways; (b) GSEA plots showing enrichment of SUMOylation of ubiquitinylation proteins and SUMOylation of RNA-binding proteins; (c,d) Quantification of Annexin A1 levels in MV_normal_ and MV_stress_ used for the corresponding SUMO1 and SUMO2/3 analyses shown in Fig. 5c; (e) WB analysis of global SUMO2/3-conjugated proteins in ADSCs after the indicated treatments with H_2_O_2_, TAK981 or SENP1-IN-3; (f) Quantification of HSPA9 interactions with Annexin A1, VDAC1 and PINK1 after pharmacological modulation of cellular SUMOylation, corresponding to Fig. 5l; (g) Quantification of Annexin A1 levels in ADSC-derived MV fractions used for the analysis shown in Fig. 5m; (h) WB analysis of Annexin A1, PINK1, VDAC1 and HSPA9 in ADSC-derived MVs under equal NTA-defined particle input after the indicated treatments; (i) Quantification of the proteins shown in h; (j) WB analysis of Annexin A1, PINK1, VDAC1 and HSPA9 in MVs normalized to equal numbers of viable donor ADSCs over the same collection period; (k) Quantification of the proteins shown in j. Quantitative data are presented as mean ± s.d. from three biological replicates obtained in three independent experiments (n = 3). Statistical significance was determined by unpaired two-tailed Student’s t-test or one-way ANOVA followed by multiple-comparisons testing, as appropriate.

**Supplementary Figure 8. Functional rescue analysis of HSPA9 K612 in mitochondrial cargo sorting and MV-induced inflammatory activation.** (a) AlphaFold predicted aligned error (PAE) map of the HSPA9 structural model used for mapping K612 in Fig. 6c. Lower predicted aligned error indicates higher relative confidence in residue positioning; (b) Quantification of HSPA9 interactions with PINK1, VDAC1 and Annexin A1 in ADSCs expressing GFP-HSPA9^WT^ or GFP-HSPA9^K612R^, corresponding to Fig. 6g; (c) WB analysis of cGAS, STING and NLRP3 in recipient NPCs treated with MVs collected from H_2_O_2_-treated ADSCs subjected to HSPA9 depletion and rescue with GFP-HSPA9^WT^ or GFP-HSPA9^K612R^. The four MV preparations were designated MV_rescue1–4_ and were derived from H_2_O_2_+si-scramble+Vector, H_2_O_2_+si-HSPA9+Vector, H_2_O_2_+si-HSPA9+GFP-HSPA9^WT^ and H_2_O_2_+si-HSPA9+GFP-HSPA9^K612R^ donor cells, respectively; (d) Quantification of cGAS, STING and NLRP3 expression shown in c; (e) WB analysis of Annexin A1, VDAC1, HSPA9 and PINK1 in MV_rescue1–4_. (f) Quantification of Annexin A1, VDAC1, HSPA9 and PINK1 expression shown in e. Quantitative data are presented as mean ± s.d. from three biological replicates obtained in three independent experiments (n = 3). Statistical significance in b was determined by unpaired two-tailed Student’s t-test; comparisons among the four rescue groups in d and f were analyzed by one-way ANOVA followed by multiple-comparisons testing.

**Supplementary Figure 9. Molecular dynamics simulation supports the structural stability of the HSPA9-PINK1 complex.** (a) Workflow of HSPA9-PINK1 complex modelling, molecular dynamics simulation and downstream structural and energetic analyses; (b) AlphaFold3-predicted HSPA9-PINK1 complex shown in surface and cartoon representations; (c) RMSD analysis of HSPA9, PINK1 and interface residues during the 100-ns molecular dynamics simulation, together with the corresponding RMSD distributions; (d–f) Superimposed conformations of HSPA9, PINK1 and the HSPA9-PINK1 complex sampled from the 40–100 ns simulation window; (g) Mapping of residue flexibility onto the HSPA9-PINK1 complex according to RMSF values; (h) Radius of gyration and corresponding distribution of HSPA9 during simulation; (i) Radius-of-gyration components of HSPA9 during simulation; (j) Radius of gyration and corresponding distribution of PINK1 during simulation; (k) Time-dependent secondary-structure evolution by residue throughout the simulation; (l) Ramachandran plot of the HSPA9-PINK1 complex; (m) Time-dependent distribution of secondary-structure elements during simulation; (n) Free-energy landscape derived from principal component analysis; (o–q) Representative initial, final and minimum-free-energy conformations of the HSPA9-PINK1 complex.

**Supplementary Figure 10. Interfacial interaction network and binding energy decomposition of the HSPA9–PINK1 complex.** (a–d) Time evolution and distribution of hydrophobic contacts, hydrogen bonds, salt bridges and π-cation interactions across the HSPA9–PINK1 interface during molecular dynamics simulation; (e) Time evolution and distribution of total interfacial interactions; (f) Residue-level interaction persistence map showing major interfacial residues and interaction types during the last 10 ns of simulation; (g) Interaction network diagram summarizing major residue pairs between HSPA9 and PINK1; (h) Total binding energy trajectory and distribution calculated by MM-GBSA; (i) Decomposition of binding energy components, including van der Waals, electrostatic, polar solvation, non-polar solvation and total energy terms; (j) Per-residue energy contribution analysis identifying interfacial hotspot residues.

**Supplementary Figure 11. Biochemical validation of direct HSPA9-PINK1 binding and orthogonal analysis of PINK1-dependent mitochondrial cargo sorting.** (a) Coomassie brilliant blue staining of purified recombinant human HSPA9 and PINK1 proteins used for SPR analysis; (b) Steady-state SPR fitting curve showing concentration-dependent binding of PINK1 to immobilized HSPA9. Steady-state fitting yielded a KD of 6.459 × 10^-7^ M; (c) Quantification of PINK1, HSPA9, VDAC1 and Annexin A1 in ADSC-derived MV fractions after PINK1 knockdown under basal or H_2_O_2_-treated conditions, corresponding to Fig. 7n. MV input was adjusted to comparable Annexin A1 levels; (d) WB analysis of PINK1, HSPA9, VDAC1 and Annexin A1 in ADSC-derived MVs under equal NTA-defined particle input after PINK1 knockdown; (e) Quantification of the proteins shown in d; (f) WB analysis of PINK1, HSPA9, VDAC1 and Annexin A1 in MVs normalized to equal numbers of viable donor ADSCs over the same collection period; (g) Quantification of the proteins shown in f; (h) Co-IP analysis of HSPA9 association with PINK1, VDAC1 and Annexin A1 after PINK1 knockdown under basal or H_2_O_2_-treated conditions; (i–k) Quantification of PINK1:HSPA9, VDAC1:HSPA9 and Annexin A1:HSPA9 interaction levels shown in h. Quantitative cellular and MV data are presented as mean ± s.d. from three biological replicates obtained in three independent experiments (n = 3). Statistical significance was determined by one-way ANOVA followed by multiple-comparisons testing.

**Supplementary Figure 12. Quantitative analyses of mitophagy regulation and orthogonal normalization of mitochondrial cargo loading after donor-cell mitochondrial quality-control modulation.** (a) Quantification of PINK1, TOMM20, HSPA9, SQSTM1, Parkin and LC3II/LC3I levels in ADSCs after HSPA9 knockdown under basal or H_2_O_2_-treated conditions, corresponding to Fig. 8a; (b) Representative line-scan fluorescence intensity profiles of TOMM20 and LC3 in ADSCs after the indicated treatments, corresponding to Fig. 8b; (c) Quantification of PINK1, TOMM20, HSPA9, SQSTM1, Parkin and LC3II/LC3I levels in ADSCs treated with H_2_O_2_, 3-MA, rapamycin (RAPA) or NMN, corresponding to Fig. 8c; (d) Quantification of the JC-1 aggregate/monomer fluorescence ratio in ADSCs after the indicated treatments, corresponding to Fig. 8d; (e) Quantification of DCFH fluorescence intensity in ADSCs after the indicated treatments, corresponding to Fig. 8f; (f) WB analysis of HSPA9, VDAC1, PINK1 and Annexin A1 in MVs under equal NTA-defined particle input after modulation of donor-cell mitochondrial quality control; (g) Quantification of the proteins shown in f; (h) WB analysis of HSPA9, VDAC1, PINK1 and Annexin A1 in MVs normalized to equal numbers of viable donor ADSCs over the same collection period; (i) Quantification of the proteins shown in h. Quantitative data are presented as mean ± s.d. from three biological replicates obtained in three independent experiments (n = 3). Statistical significance was determined by one-way ANOVA followed by multiple-comparisons testing.

**Supplementary Figure 13. Quantitative validation of donor-cell mitochondrial rescue and the therapeutic effects of MVmodified.** (a) Quantification of mitochondrial membrane potential by JC-1 analysis in ADSCs treated with H_2_O_2_, NMN, si-scramble, si-HSPA9 or the indicated combinations, corresponding to Fig. 9a; (b) Representative DCFH-DA fluorescence images showing intracellular ROS accumulation in ADSCs after the indicated treatments. α-Tubulin and DAPI were used to visualize the cytoskeleton and nuclei, respectively. Scale bar, 20 μm; (c) Quantification of DCFH-DA fluorescence intensity shown in b; (d) Quantification of cGAS, STING and NLRP3 expression in H_2_O_2_-injured NPCs treated with MVnormal, MVstress or MVmodified, corresponding to Fig. 9g; (e) Histological scores of rat caudal discs in the IVDD, MVnormal, MVstress and MVmodified groups; (f) Representative line-scan fluorescence intensity profiles of cGAS and STING in rat disc tissues from the IVDD, MV_normal_, MV_stress_ and MV_modified_ groups, corresponding to Fig. 9p. For in vitro experiments, data are presented as mean ± s.d. from three biological replicates obtained in three independent experiments (n = 3). For animal experiments, n = 5 rats per group, with one treated disc per rat considered one independent biological replicate. Statistical significance was determined by one-way ANOVA followed by multiple-comparisons testing.
